# Clinical Guidelines for Hepatitis E Vaccination in India: An Expert Panel Consensus Report on the Recombinant Hepatitis E Vaccine, HEV 239

**DOI:** 10.3390/pathogens15080783

**Published:** 2026-07-23

**Authors:** Mohammad Sultan Khuroo, Naira S. Khuroo

**Affiliations:** Digestive Diseases Center, Dr. Khuroo’s Medical Clinic, Kashmir, Srinagar 190010, India; naira_sultan@yahoo.com

**Keywords:** hepatitis E, hepatitis E virus, genotypes, HEV 239, Hecolin^®^ India, modified Delphi process, expert consensus, clinical guidelines, epidemics, chronic liver disease, pregnancy, acute-on-chronic liver failure, solid organ transplantation, people living with HIV, targeted vaccination

## Abstract

(1) Background: Hepatitis E remains a major public health challenge in India. (2) Methods: In August 2025, the recombinant HEV 239 vaccine was approved in India for adults aged 18 to 65 years. To establish clinical guidelines tailored to the Indian setting, an expert panel consensus was conducted using a modified Delphi process in accordance with the ACCORD reporting guidelines. (3) Results: A steering committee put forth 18 statements covering vaccine safety, efficacy, and clinical indications, which were independently evaluated by 33 senior Indian hepatologists and epidemiologists. Consensus was assessed using the GRADE framework for level of evidence, balance of benefits and harms, and strength of recommendations. Of the 18 statements, 12 reached the 70% consensus threshold. The panel concluded that the vaccine, which is administered on a standard three-dose schedule, is safe and highly effective in healthy adults, providing protection for up to 10 years. Targeted vaccination was recommended for five high-risk populations: outbreak-affected groups, hyperendemic pockets, women of childbearing age, patients with CLD, and solid organ transplant recipients. Although derived from HEV genotype 1, the vaccine demonstrated cross-protective efficacy against HEV genotype 4. (4) Conclusions: This consensus report provides a framework for deploying the HEV vaccine to mitigate disease burden in India while emphasizing the need for real-world effectiveness and safety data from India.

## 1. Introduction

Hepatitis E is a globally important infectious disease caused by the hepatitis E virus (HEV) [1]. HEV belongs to the family *Hepeviridae*, which comprises two subfamilies, five genera, and ten species that infect a wide range of vertebrate hosts [2]. Four HEV genotypes (HEV-gts) in the genus *Paslahepevirus* (*Orthohepevirus A*) infect humans. HEV-gt1 and HEV-gt2 are anthroponotic and are transmitted exclusively between humans [1,3]. HEV-gt3 and HEV-gt4 are enzootic, infecting pigs and some other animals, and may be transmitted to humans through specific foodborne zoonotic transmission pathways [4]. Finally, Rat HEV and Camel HEV can infect solid organ transplant (SOT) recipients [5,6,7].

Hepatitis E was first recognized during an epidemic of non-A, non-B hepatitis in Kashmir, India, in 1980 [8]. Balayan et al., via self-experimentation, visualized virus-like particles (VLPs) in stool samples [9]. More recently, researchers at Gene Lab USA successfully isolated, cloned, and sequenced the full-length genome of the HEV [10,11,12]. Over the last four decades, major milestones have been achieved in hepatitis E research [13].

Hepatitis E has emerged as an important global health concern. Global seroprevalence data indicate that approximately 12.5% of the population, nearly 939 million, have had prior HEV exposure. Of these individuals, active or persistent infections continue to impact between 15 and 110 million people [14]. Geographic patterns of hepatitis E vary between developing and developed nations and are largely determined by the circulating HEV-gts. While HEV-gt1 is widespread across Africa and Asia, HEV-gt2 is notably more geographically restricted, occurring primarily in Mexico and West Africa [15]. Transmission of HEV-gt1 and HEV-gt2 occurs through the fecal-oral route, typically due to inadequate sanitation and contaminated water. In hyperendemic regions of Asia and Africa, seroprevalence rates range from 7% to 30% [14]. These regions face a substantial public health burden, with an estimated 3.3 million clinical cases, 70,000 deaths, and 3000 stillbirths occurring annually [16].

HEV-gt3 is found in Europe, North America, parts of Asia, Oceania, several African countries, and South American countries. In contrast, HEV-gt4 is predominantly found in China, Korea, and Japan, with limited circulation in France and Italy [1,17,18] HEV-gt3 and HEV-gt4 are increasingly recognized as zoonotic pathogens of concern [4]. Pigs, among other animals, are a significant reservoir of HEV. Human infection occurs through eating undercooked pork liver (sausages) or game (wild boar and deer), direct contact with infected animals (mainly pigs), contaminated water from slurry, or, less commonly, blood transfusions [19,20]. Infections are responsible for the vast majority of autochthonous (locally acquired) hepatitis E cases in industrialized nations, particularly in Europe (HEV-gt3) and Asia (HEV-gt4) [18,21]. HEV-gt3 can cause chronic hepatitis, fibrosis, and cirrhosis in patients with solid organ transplants (SOTs), PLWH (people living with HIV), and hematopoietic neoplasms [22,23]. The burden of disease in SOT recipients is high (around 20%), with liver transplant patients at the highest risk (27.2%), followed by renal/heart transplant (12.8%) and lung transplant (5.6%) patients [24].

Four recombinant hepatitis E vaccines have reached the clinical trial phase [25]. The recombinant HEV 239 (Hecolin^®^) is currently the only vaccine available for clinical use and has been marketed in China since October 2012 [26]. Based on the results of a phase III trial, the vaccine received approval for use on Indian subjects aged 18 to 65 years in August 2025 and has been marketed by Urihk Pharmaceuticals under the brand name Hevrevac^®^ and by Dr. Reddy’s Laboratories under the brand name Hevaxin^®^ [27].

The vaccine is an effective means of fighting hepatitis E, a disease of significant public health importance in India [26]. However, most clinical trials for the hepatitis E vaccine have been conducted in China, where hepatitis E epidemiology differs. Additionally, changes in the epidemiology of hepatitis E in India necessitate targeted vaccination in high-risk groups first. This requires developing consensus on the use of the hepatitis E vaccine in India, which would provide a framework for deploying the HEV vaccine to mitigate disease burden and generate real-world data for the development of guidelines for its use.

## 2. Methodology

We conducted an expert panel consensus on the use of HEV 239 (Hecolin^®^) in India using a modified Delphi process [28]. Unlike a traditional Delphi process that starts with open-ended questions, a modified Delphi usually begins with a literature review and a pre-defined set of statements derived from that evidence [29,30]. ACCORD (Accurate Consensus Reporting Document) guidelines were followed throughout the process, including during consensus development [31]. The process followed five stages, as defined by the EQUATOR (Enhancing the QUAlity and Transparency Of health Research) Network guidelines, including establishing a steering committee, developing a document with a set of statements derived from a critical literature review, using a modified Delphi process for consensus, developing a consensus report, and publication [32].

**Figure 1:**
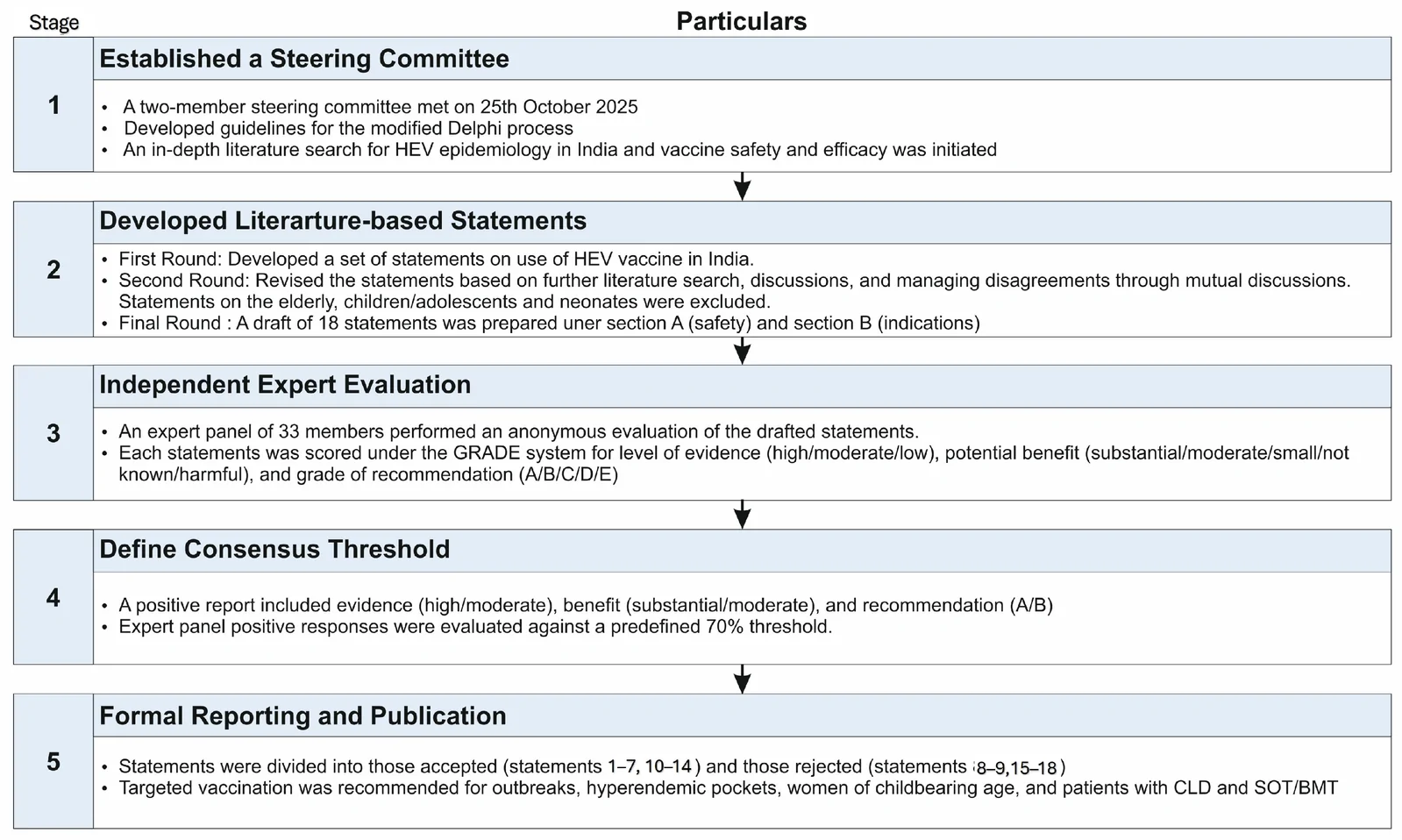
Flow diagram depicting five stages followed during consensus building for use of HEV 239 (Hecolin^®^) in India. [GRADE = Grading of Recommendations, Assessment, Development, and Evaluation; CLD = chronic liver disease; SOT = Solid Organ Transplants; BMT = bone marrow transplant].

Two of the authors of this article (MSK and NSK) constituted the steering committee that led and coordinated the guideline development process. (Figure 1) The committee met for the first time in Srinagar on 25 October 2025 and subsequently held three more sessions. We both independently conducted an in-depth literature search on the current status of hepatitis E infection in India, including the local disease burden, prevalent genotypes in humans and animals, epidemiology, transmission routes, disease patterns, and future projections. A critical review of the vaccine’s safety, immunogenicity, and effectiveness was synthesized from data from well-conducted published studies. Based on the outcomes, we drafted a set of statements regarding the use of HEV 239 (Hecolin^®^) in India and discussed them in subsequent meetings. All disagreements were resolved by mutual discussion. The statements were revised based on further literature searches, the expert panel’s opinion, and the resolution of pending disagreements. The statements on the use of the vaccine in the elderly, children/adolescents, and neonates were dropped from the original list. Finally, we generated a document containing 18 statements of fact about HEV and the hepatitis E vaccination in India.

The statements were divided into two sections. Section A outlined the safety, immunogenicity, efficacy, and different dosing schedules of HEV 239 (Hecolin^®^), and Section B covered the potential indications for its use in India. Each statement included a brief review of well-conducted published studies, two to three important abstracts, and well-designed explanatory data tables. This document was reviewed by the expert panel and, based on their inputs, was used to generate a consensus report.

The expert panel was carefully selected from among senior practicing hepatologists (>5 year experience) at academic institutions across India [28]. Individuals in leadership roles (e.g., program directors, clinical heads) and with a research background were preferably selected. We believed that an expert team of 30 or more members would be optimal for generating a broad consensus. Major Indian academic institutions, both governmental and private, were identified, and 50 senior practicing hepatologists (after accounting for dropout) were randomly selected. The panelists were personally invited to participate in the consensus. Panelists with conflicts of interest, as determined by voluntary disclosure, were excluded. No incentives were used to encourage responses or participation in the consensus process. The steering committee members were not included in the expert panel. Once participants consented, the document containing 18 statements was mailed to each of them, along with a cover letter explaining the expert panel’s consensus process. A 4-week period was given for filing the report. During the review, the panelists were free to ask questions and request the full versions of any articles cited in the document. The steering committee members did not attempt to influence the expert panel on the use of the HEV 239 (Hecolin^®^) in India. The consensus process maintained strict anonymity, and the experts submitted their evaluations to the steering committee independently, without knowing who else was on the panel. The expert panelists who participated in the consensus are acknowledged, in accordance with COPE (Committee on Publication Ethics) and ICMJE (International Committee of Medical Journal Editors) guidelines [33].

The experts were required to address each statement using the GRADE system (Grading of Recommendations Assessment, Development and Evaluation) and divide their response into levels of evidence, potential benefits, and grades of recommendation (Table 1) [34]. Thus, for each statement, the expert panel could select three options from the checklist: one each for the level of evidence (high, moderate, or low), potential benefits (substantial, moderate, small, not known, or harmful), and the grade of recommendation (A to E). The responses were loaded onto an Excel sheet and evaluated jointly by the steering committee. We set 70% as the consensus threshold for accepting evidence, benefits, and recommendations. A positive report on the vaccine had to include a level of evidence of high or moderate, a potential benefit of substantial or moderate, and recommendations of A or B.

## 3. Result

The consensus document, containing 18 statements, was sent to 50 expert panelists. We received responses from 33 (66%) panelists. Other expert members were not persuaded to participate after we crossed the thirty-member threshold. Twenty-six participating experts were practicing gastroenterologists/hepatologists: three practiced exclusively hepatology, two were surgical gastroenterologists, and two were epidemiologists with a special interest in viral hepatitis. The panelists had 19.02 ± 8.82 years of clinical experience (95% CI, 16.17–21.86 years). Twenty-four were faculty members at tertiary corporate hospitals, while nine worked at governmental academic institutions. Seven experts held the positions of Director/Associate Director/Chairman/Head of their respective departments, while the other 26 served as senior consultants. The experts were working in ten Indian states/Union territories: Maharashtra, Uttar Pradesh, Haryana, New Delhi, Jammu and Kashmir, Karnataka, West Bengal, Gujarat, Uttarakhand, and Tamil Nadu. The expert panel members are acknowledged [33,35].

It is important to note that the vaccination mentioned in the statements below refers to the use of the recombinant hepatitis E vaccine, HEV 239 (Hecolin^®^) on a three-dose schedule (0, 1, and 6 months), unless specified otherwise. The same drug was launched and is marketed in India under different brand names.

Section A: The safety, immunogenicity, efficacy, and dosage schedule of the HEV 239 (Hecolin^®^).

**Figure 2:**
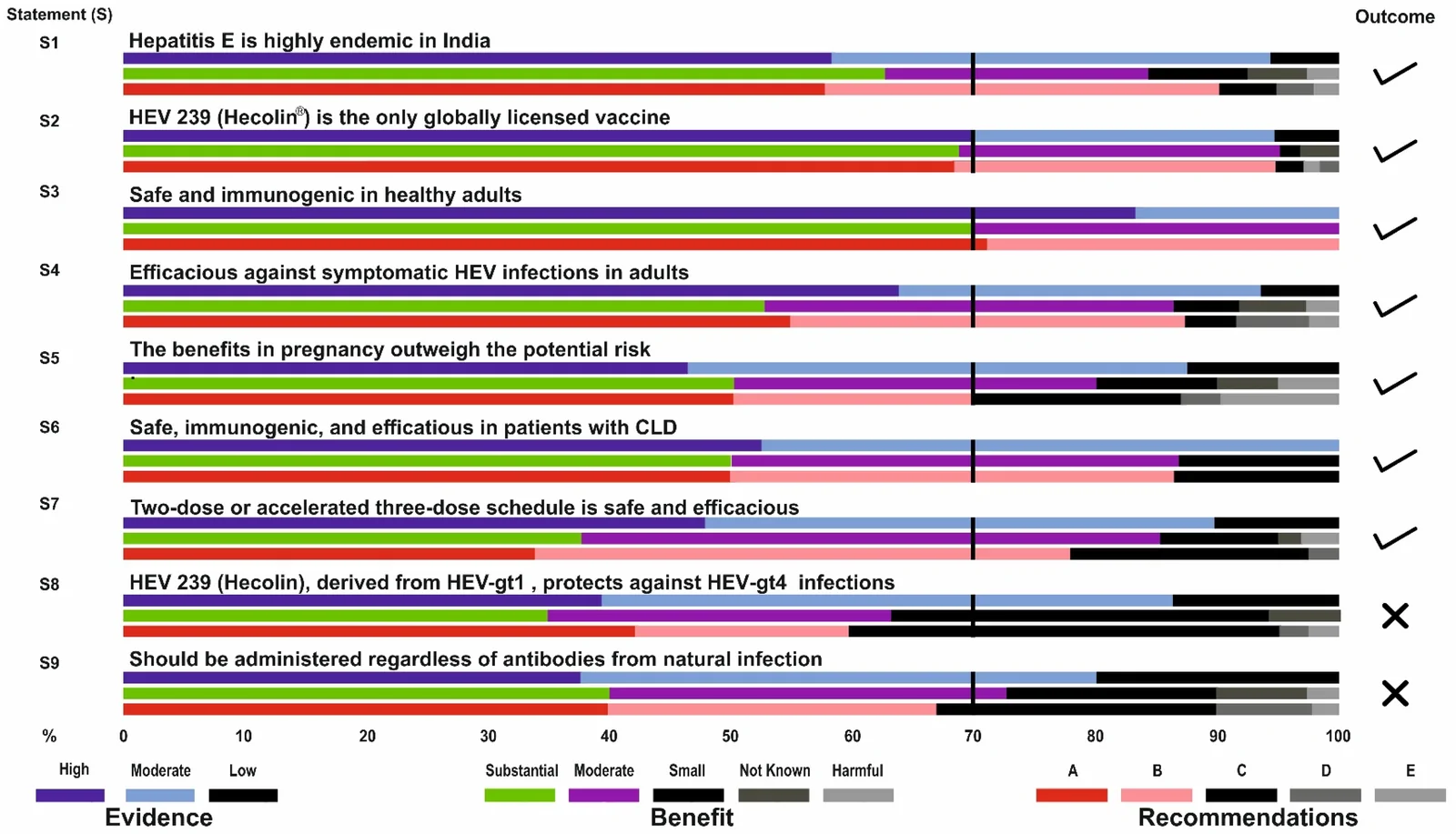
The cumulative response to the nine statements in Section A by the expert panel. The responses were based on the GRADE system. We set 70% as the consensus threshold for accepting evidence, benefits, and recommendations (vertical bars). A positive report on the vaccine had to include a level of evidence of high or moderate, a potential benefit of substantial or moderate, and recommendations of A or B. Statements 1 to 7 were accepted (marked ✓ under outcome), while statements 8 and 9 were rejected (marked ✕ under outcome).

Nine statements in Section A (numbers 1 to 9) were evaluated by the expert panel for their level of evidence, potential benefits, and recommendations. The cumulative responses of the expert panel to each statement are shown in Figure 2.

**Statement 1.** Hepatitis E is highly endemic in India, with a recent demographic and clinical transition. Healthcare providers should consider using the hepatitis E vaccine for proven indications.

Consensus: evidence, moderate; benefit, moderate; recommendation, B.

HEV infection is a pervasive, endemic health issue among India’s 1.4 billion people [36,37,38]. Existing epidemiological assessments report an age-adjusted prevalence and incidence of 29.91 and 433.01 per 100,000, respectively, with a disability-adjusted life year metric of 9.52 [39]. However, these figures underrepresent its true impact due to systemic underdiagnosis and underreporting [40]. Despite regional declines, India, along with Bangladesh, consistently ranks among the countries with the highest acute hepatitis E incidence and disability-adjusted life years (DALYs) [39,41,42,43].

The disease manifests as repeated waterborne epidemics causing death and devastation [44]. In addition, HEV continues to drive seasonal, localized outbreaks, often during and after monsoons, due to sewage-contaminated water supplies [45,46,47]. Hepatitis E is the most common cause of acute sporadic viral hepatitis and acute liver failure in India [36,37,48,49,50,51]. HEV remains a lethal threat to pregnant women, particularly in the third trimester, as it often progresses to acute liver failure with high fatality rates [52]. The incidence and severity of the disease are increased in pregnant women, causing poor obstetric outcomes such as stillbirths and premature deliveries [52,53,54]. It also poses a severe risk to individuals with pre-existing chronic liver disease [55]. HEV acts as a major trigger (ranging from 7 to 60%) for acute-on-chronic liver failure (ACLF) in patients with chronic hepatitis B cirrhosis or other liver pathologies [56,57,58,59]. ACLF is defined by a precipitous loss of hepatic performance and multi-organ dysfunction, carrying a stark 28-day mortality risk of nearly 15%. This condition accounts for roughly one-third of clinical admissions for decompensated cirrhosis [60]. Patients with cirrhosis face an estimated 40% cumulative likelihood of experiencing ACLF over a ten-year horizon [61].

Over the last decade, the epidemiology of HAV and HEV in India has undergone a profound demographic and clinical transition [62,63,64]. Seroprevalence surveys in children have steadily declined, leaving a large population of adolescents and young adults who lack protective antibodies undetected [65]. Hepatitis A disease in adults is much more severe, often presenting as acute liver failure [66,67]. At the same time, hospital admissions and laboratory-confirmed cases of HEV have declined overall. For example, a 5-year trend analysis (2018–2022) at a tertiary care center in South Gujarat showed that HEV positivity declined by 64.5%, suggesting reduced transmission of sporadic cases [68]. The overall burden and test positivity rates for HEV are declining in parts of India [36]. While epidemics have become less common, outbreaks of hepatitis E are a frequent occurrence [45,46]. Moreover, hepatitis E has transformed into a more endemic, sporadic disease [68].

The changing epidemiology of the two enteric hepatitis viruses in India could be due to significant changes in socio-economic status and the launch of Swachh Bharat Abhiyan (the nationwide sanitation campaign) on 2 October 2014, which fundamentally altered the sanitation landscape in India [69,70]. Sanitation coverage increased from below 40% in 2014 to near-universal (99%), effectively eliminating open defecation and significantly improving public health outcomes and economic loss ratios. Efforts to address groundwater depletion and promote equitable water resource management are ongoing [71]. Thus, we believe that an improved water, sanitation, and hygiene (WASH) infrastructure could have had a significant impact on the epidemiology of HAV and HEV in India [70]. The changing epidemiology of HEV could also reflect a cohort phenomenon. With significant exposure over the years, the vast majority of adults and adolescents acquire natural immunity. This leads to a significant drop in the disease burden for decades. Years later, a new, susceptible generation is born, and the disease reappears in the community, in the form of either repeated epidemics or sporadic cases [72,73].

The burden of human hepatitis E in India is exclusively the result of HEV-gt1 [74]. HEV-gt1 predominates among human infections, but HEV-gt4 is widespread in Indian pigs [75]. This raises the possibility of cross-species transmission to humans, as has occurred in China over the last two decades [76,77]. HEV is a significant emergent zoonotic pathogen, with HEV-gt3 and HEV-gt4 ranking amongst the most common zoonotic viral infections. HEV ranks sixth in terms of global pandemic potential, following the Lassa, SARS-CoV-2, Ebola, Saoul, and Nipah viruses [78]. However, most humans contract HEV by eating raw or undercooked pork and liver [79]. The risk of a widespread zoonotic outbreak in India, replicating the Chinese case, is currently mitigated by the population’s dietary habits. In India, pork consumption is relatively low compared to in China, and pork is typically cooked at high temperatures, which easily inactivate the virus [80]. Direct contact with infected animals can transmit HEV, increasing the risk to veterinarians, farmers, and other animal handlers [81].

The disease is vaccine-preventable [82]. We recommend using the vaccine for proven indications to relieve human suffering, save lives, and mitigate the global impact [83]. However, in view of the changing epidemiology of hepatitis E in India, vaccination may initially be restricted to high-risk groups.

**Statement 2.** Four hepatitis E vaccines have entered the arena of human experimentation. HEV-239 vaccine is the only globally licensed vaccine at present.

Consensus: evidence, moderate; benefit, moderate; recommendation, B.

Culturing HEV in vitro is challenging and inefficient due to low viral titers and slow replication rates [84]. The lack of an efficient in vitro culture system is one of the biggest challenges in HEV research. The use of traditional live or attenuated vaccines is not feasible as they require high-volume cultivation, making recombinant subunit vaccine technology the only successful approach [85]. The existing HEV vaccines are based on recombinant technology, utilizing a truncated form of the viral capsid protein to produce virus-like particles (VLPs) [86,87].

HEV is a small, spherical virus with icosahedral symmetry that exists in quasi-enveloped (qeHEV, 40 nm) and non-enveloped, naked (neHEV, 27–34 nm) forms [88,89,90]. Its capsid encloses a positive-sense, single-stranded RNA genome (+ssRNA) of 7.2 kb. HEV RNA contains three open reading frames (ORFs), with an additional ORF4 found in HEV-gt1. HEV RNA is translated into a non-structural polyprotein (ORF1, aa 1693) with eight domains. One such domain (RNA-dependent RNA polymerase) uses genomic RNA to produce a full-length negative-sense RNA, which serves as a template for full-length genomic RNA and a 2.2 kb subgenomic RNA (sgRNA). sgRNA encodes the ORF2 protein (aa 660) and the multifunctional ORF3 protein, which acts as an egress protein (aa 114).

The HEV capsid has an icosahedral structure with a triangulation number T = 3 and is composed of 180 protein subunits encoded by ORF2 (aa 660) [91,92]. It consists of an N-terminal signal peptide (aa 1–23), N-terminus (aa 14–111), three distinct linear domains (S, M, and P), and a C-terminus (aa 608–660). The S domain (aa 118–313) forms a continuous icosahedral shell, providing the capsid’s structural integrity and enabling self-assembly. The M domain (aa 314–453) bridges the S and P domains, helps stabilize the capsid structure, forms three-fold protrusions, and participates in receptor binding. The P domain (aa 454–608) forms two-fold dimeric spikes on the surface, is highly antigenic, and contains the virus’s neutralizing epitopes, thereby playing a key role in immune recognition [17,93,94,95].

Hepatitis E vaccine development is based on the assembly process of the HEV capsid [86,87,93,96]. Due to variations in ORF2 components, there are significant differences in viral expression and assembly [91]. Located adjacent to and overlapping the signal peptide is an arginine-rich motif (ARM, a five-amino-acid sequence), which acts as a switch for ORF2 to be secreted as an immune decoy (ORF2S) [97,98]. The adjacent N-terminus (aa 14–111) contains a key domain responsible for binding the 5′ end of the HEV RNA for viral encapsulation (T = 3). The interaction between the N-terminus and HEV-RNA forces the capsid protein into a flat dimer configuration, preventing premature T = 1 (triangulation number) formation and the formation of the native, infectious T = 3 virion. Thus, the HEV virion (T = 3) is large (320–340 Å), with 180 subunits, and includes the N-terminal arginine-rich domain for assembly and RNA packaging.

If this N-terminal region is truncated (e.g., in laboratory expression), the capsid protein subunits adopt a curved conformation, thereby forcing assembly into T = 1 symmetry [86]. HEV VLPs (T = 1) are structurally truncated, smaller versions of the native virions (270 Å), composed of 60 capsid protein subunits and lacking the N-terminal arginine-rich region required for RNA encapsidation, thereby rendering them inherently distinct from native infectious particles in both assembly mechanisms and physical stability. HEV VLP (T = 1) retains the key antigenic P-domain (aa 456–606 of ORF2 capsid) that elicits neutralizing antibodies and is more efficient to produce and purify, making it a better subunit vaccine platform than native (T = 3) particles [86,91,93].

Four hepatitis E vaccines based on ORF2 expression and self-assembly of VLPs with a neutralizing epitope (aa 459–606 ORF2 capsid) have entered the clinical trial phase (Table 2) [93,96,99]. One of them, rHEV 56 kDa (VLP, T = 1, 270Å, Glaxo, HEV-gt1, aa 112–607 ORF2), has passed a phase III trial in Nepal and has been shown to be safe, immunogenic, and efficacious [100]. However, further progress in its licensing and commercial production was stalled due to economic/commercial factors [101]. Two additional vaccines, namely HEV-179 (VLP, T = 1, 120Å, Changchun Institute of Biological Products, HEV-gt4, aa 439–617 ORF2) and ZyVacHEV (Lipo-NE-P, Zydus, HEV-gt1, aa 458–607 ORF2), have been shown to be safe and immunogenic in early-phase I and phase II trials [102,103]. However, neither has yet undergone a phase III (efficacy) trial, and neither is currently licensed for human use in any country.

The HEV 239 Hecolin^®^ (VLP, T = 1, 230 Å, Xiamen Innovax Biotech Co., Ltd., Xiamen, China, HEV-gt1, aa 368–606 ORF2 capsid) has been licensed in China (2012), Pakistan (2021), and India (2025) [101,104]. Attempts to approve this vaccine in other countries, including Bangladesh, are underway [105]. The vaccine is based on ORF2, which encodes the 660-amino acid major structural capsid protein. The antigen consists of a truncated fragment of the ORF2 protein, specifically amino acids 368–606. This fragment (known as HEV-239) is engineered to self-assemble into 23 nm diameter T = 1 icosahedral VLPs. The protein is expressed in *Escherichia coli* (*E. coli*), enabling easy upscaling and high-efficiency production. The purified VLP antigen is adsorbed onto an aluminum hydroxide adjuvant to boost the immune response. The design focuses on the P2 domain (amino acids 454–606) of the capsid, which mediates host interactions and contains major neutralizing epitopes (specifically identified at residues such as Arg512). Although the HEV 239 is derived from HEV-gt1, it provides cross-protection against HEV-gt4 because all human HEV-gts share a single serotype. As VLPs do not contain viral nucleic acid, the vaccine is non-infectious and safe. The particulate form of the VLP is significantly more immunogenic than the free recombinant protein. The vaccine is stable for 36 months when stored at 2–8 °C. The licensed vaccine, Hecolin^®^, is administered on a three-dose schedule (0, 1, and 6 months) and has demonstrated >95% efficacy.

**Table 2:** Hepatitis E vaccine under clinical evaluation.

Vaccine	Manufacturer	Antigen	Express System	Structure	Dose	Efficacy	Development Status	References
**HEV 239 (Hecolin)**	Xiamen Innovax Biotech Co., Ltd., Xiamen, Fujian, China	HEV-1 ORF2 (aa 368–606)	*Escherichia coli*	VLP T = 1 Icosahedral 230 Å Empty	0, 1, 6 m	100% (95% CI 72.1–100.0)	Licensed * (China, Pakistan, India **#**) Phase IV	[106]
**rHEV** **56 kDa**	GlaxoSmithKline (Brentford, UK) NIH Bethesda, MD, USA	HEV-1 ORF2 (aa 112–607)	*Baculovirus*	VLP T = 1 Icosahedral 270 Å 60 capsids Empty	0, 1, 6 m	95.5% (95% CI 85.6–98.6)	Phase III No further progress	[100]
Lipo-NE-P ** **(ZyVacHEV)**	Zydus Lifesciences Ltd. (Ahmedabad, India)	HEV-1 ORF2 (aa 458–607)	*Escherichia coli*	NE Protein encapsulated within liposomes ***	0, 1, 6 m	Phase 1 results revealed vaccine safety and tolerance	Phase II (Under progress)	[103]
**HEV P179**	Changchun Institute of Biological Products Co., Ltd. Changchun, China	HEV-4 ORF2 (aa 439–617)	*Escherichia coli*	VLP T = 1 Icosahedral 120 Å Empty	0, 1, 6 m	Phase 1 results revealed vaccine safety and tolerance	Phase Ib	[102]

* = not prequalified by WHO; however, it recommends its use in outbreaks. Registration is in progress in Bangladesh, Nepal, and Indonesia. ** = Liposome-encapsulated neutralizing epitope protein. *** = NE protein is loaded into the aqueous core of the liposome. Liposomes protect the antigen, facilitate targeted delivery, and exert an adjuvant effect. # = marketed as Hevrevac (URIHK Pharmaceuticals Pvt. Ltd., Mumbai, India).

**Statement 3**. HEV-239 (Hecolin^®^) is safe and immunogenic in healthy adults when administered on a three-dose schedule of 0.5 mL (30 µg) each.

Consensus: evidence, high; benefit, substantial; recommendation, A.

HEV 239 (Hecolin^®^) has undergone extensive phase I and II studies among healthy adults in China, Bangladesh, the USA, and India (Table 3) [106,107,108,109]. Phase I clinical trials demonstrated that it is safe and well-tolerated. The most frequently reported reactions are mild, including local pain and swelling at the injection site, as well as systemic symptoms such as malaise, fatigue, headache, and myalgia. The US Phase 1 trial reported that 85% of subjects experienced systemic events, with headache the most common (55%). Importantly, all adverse events were Grade I (mild) or Grade 2 (moderate) in severity, and there were no serious adverse events (SAEs). Some participants reported unsolicited adverse events (such as elevated body temperature or nausea), but these were considered unrelated to the vaccine. Laboratory abnormalities were mostly mild and resolved at follow-up, with no significant differences from the placebo group. In summary, the adverse reactions observed were consistent with typical vaccination protocols, with no high-grade or safety-related serious events associated with the vaccine.

Immunogenicity was reported in both phase I and phase II studies. The phase II study was conducted in two parts: a dose-scheduling component to determine the optimal number of vaccine doses and a dose-escalation component to determine the optimal dose. Seroconversion was achieved in 98% and 100% of subjects after two and three vaccine doses, respectively, compared with only 8% in the control group [107].

**Statement 4.** The hepatitis E vaccine is efficacious against symptomatic HEV infections in healthy adults.

Consensus: evidence, moderate; benefit, moderate; recommendation, B.

The HEV-239 vaccine was used in a large-scale (112,000 healthy adults), randomized, double-blind, placebo-controlled, phase III trial (ClinicalTrials.gov, number NCT01014845) against symptomatic HEV infection conducted in Dongtai County, Jiangsu, China. (Table 4) Participants were randomly assigned to a vaccine *group (n = 56,302) or a placebo group (n = 56,302)*, and 86% of participants in both groups received three doses and were included in the primary efficacy analysis. The initial results reported (12 months) demonstrated 100% vaccine efficacy (fifteen HEV cases in the placebo group versus none in the vaccine group) against confirmed hepatitis E among participants who received all three doses and a 100% immune response [111]. At the 4.5-year follow-up, the vaccine showed 86.8% efficacy (53 HEV cases in the placebo group versus 3 in the vaccine group), with a sustained immune response in 87% of vaccinees [111]. At the 10-year follow-up, the vaccine showed 83.1% efficacy (77 HEV cases in the placebo group versus 13 in the vaccine group), with a sustained immune response in 87.3% of vaccinees at 8.5 years [112]. This trial demonstrated that HEV239 was well tolerated and effective in preventing hepatitis E in the general population in China, including both men and women aged 16–65 years. These trials confirmed the long-term efficacy of HEV 239 in healthy adults from China. They provide strong evidence to support the promotion and implementation of hepatitis E vaccination, particularly in high-endemic zones.

**Statement 5.** The benefits of using the HEV vaccine during pregnancy outweigh the potential risks.

Consensus: evidence, moderate; benefit, moderate; recommendation, B.

HEV-239 (Hecolin^®^) has been used in women during pregnancy in Bangladesh and South Sudan (Table 5) [114,115,116,117]. A preliminary study from China demonstrated the safety of the hepatitis E vaccine during pregnancy [118]. Studies from Bangladesh have revealed a potentially increased risk of miscarriage when the vaccine is administered in the 90 days before conception or early in the first trimester. Pregnant individuals were excluded from clinical trials, so safety data were derived from post hoc analyses and observational studies, highlighting a knowledge gap. No increased risk was observed for stillbirth or elective termination, nor for women vaccinated more than 90 days before pregnancy [115]. Data from large vaccination campaigns in South Sudan (Bentiu) and related studies using the HEV-239 vaccine show no increased risk of fetal loss with vaccination during pregnancy. A post hoc analysis of a randomized, double-blind HPV trial in which Hecolin^®^ was used in the control arm was conducted. During this trial, 140 pregnant women were inadvertently included. The incidence of adverse events in pregnant women who received either Hecolin or the HPV vaccine (Cecolin) was similar (31.8% vs. 35.1%, *p*  =  0.6782). Hepatitis E vaccination during or shortly before pregnancy was not associated with increased risks for either the pregnant woman or pregnancy outcomes [119]. The WHO’s Strategic Advisory Group of Experts (SAGE) and the Global Advisory Committee on Vaccine Safety (GACVS) reviewed HEV vaccination during pregnancy. The committee concluded that the benefits of HEV vaccination in pregnancy outweigh risks in vulnerable areas with circulating HEV, especially for women of childbearing age. We discussed the evidence from multiple publications, reviews, and editorials regarding the safety of HEV-239 during pregnancy. Despite several trials demonstrating its safety profile, data published from Bangladesh warrant serious consideration. At present, the use of HEV 239 in pregnant women in India is not permitted by the regulatory body [106,116,120,121].

**Statement 6.** The hepatitis E vaccine is safe, immunogenic, and efficacious in patients with chronic liver disease, including those with compensated cirrhosis and chronic HBV infection.

Consensus: evidence, moderate; benefit, moderate; recommendation, B.

The HEV-239 is safe and highly immunogenic for patients with chronic liver disease (CLD), including those with compensated cirrhosis and chronic hepatitis B virus (HBV) infection (Table 6) [122]. Most reported adverse events are mild and comparable to those in the general population. The vaccine does not significantly worsen liver function, and liver indicators generally remain stable or may improve with vaccination. All participants seroconverted with a robust antibody response in over 82% within one month, a rate comparable to that of healthy adults. There is a significantly reduced risk of medically attended symptomatic hepatitis E, with an estimated effectiveness of 72% [123]. By preventing acute HEV superinfection in such patients, the vaccine effectively prevents potentially fatal acute-on-chronic liver failure [124]. Data on the use of HEV vaccine in patients with decompensated cirrhosis are less robust. Recommendations for the use of the vaccine in such patients need to be based on the risk of HEV infection and potentially reduced, shorter-duration immune responses.

**Statement 7**. A two-dose or accelerated three-dose schedule of HEV 239 (Hecolin^®^) can be used safely when a rapid antibody response is required.

Consensus: evidence, moderate; benefit, moderate; recommendation, B.

HEV 239 is safe, immunogenic, and efficacious when administered on a three-dose schedule (0, 1, and 6 months). However, two doses (at 0 and 1 months) or an accelerated three-dose schedule (at 0, 7, and 21 days) are also safe and result in rapid seroconversion with significant short-term (up to 13 months) protection (Table 7) [125,126,127]. A single dose of Hecolin^®^ shows moderate short-term effectiveness, ranging from 72.1 to 86.6%.

Prior serological status determines the antibody response, which it is robust among those who are seropositive at baseline and low and short-lasting among seronegative individuals [123]. Studies during an outbreak in South Sudan showed a two-dose effectiveness of approximately 89.4% against HEV-gt1 [128]. The committee recommends using these dosage schedules when rapid protection is required, such as during HEV outbreaks, among travelers to endemic areas, and among persons exposed or likely to be exposed to the virus.

**Table 7:** HEV 239 (Hecolin^®^): two-dose and accelerated dose schedules.

Phase	Region	Trial Objectives	Design	Dosage Schedule	No of Subjects	ADRs	Immune Response	Results	Conclusion	Reference
III	China (2019)	Accelerated schedule	Random allocation (1:1); serial antibody response	Accelerated (Day 0, 7 & 21) vs. routine (0, 1 & 6 months)	126 (63 vs. 63)	32.26% vs. 30.18% (NS); most moderate AEs, no SAEs.	Both groups 100%; GMC ratio, 0.88	-	An accelerated schedule is safe and provides protective antibodies more quickly than the routine schedule	[129]
III	China (2024)	Different dose schedules		Phase 3 trial (extended)		Single dose: 49 of 50 with negative baseline seroconverted, and 9 of 18 maintained antibodies at 91 months.	Two doses: 200 of 202 with negative baseline seroconverted and 71 of 121 maintained antibodies at 91 months	Seropositive individuals had prolonged and higher antibody responses	Both single and two-dose regimens demonstrated notable immunogenicity and persistence	[127]
III	Bangladesh (2023)	Two-dose schedule	Random allocation to HEV vaccine & HBV Vaccine; test at 0, 60 days, and 2 years	1 & 2 months	100 healthy subjects (16–39 years)	Mild, comparable	All subjects who received the HEV vaccine seroconverted, with robust and long-lasting seroconversion	-	The two-dose regimen is safe and produces a long-lasting functional immune response	[126]
**IV**	Bentiu, South Sudan (2025)	Two-dose efficacy	Primary: case–control study (1:6); Secondary: test-negative design; protracted outbreaks	Two-dose regimen (0 & 1 month)	Refugee camp residents 16–40 years	-	-	21 HEV infections; 10 unvaccinated compared with 33 of the 121 matched controls (effectiveness CC 84%%; test negative: 89.4%)	A two-dose regimen is effective against the hepatitis epidemic	[130]

**Statement 8.** HEV 239 (Hecolin^®^), derived from HEV-gt1, shows efficacy against HEV-gt4.

Consensus: evidence: moderate; benefit, moderate; recommendation, B.

Current hepatitis E vaccines are produced from a single genotype. Three HEV vaccines, rHEV, HEV239, and ZyVacHEV, are derived from HEV-gt1 and the HEV 179 from HEV-gt4 [131]. Do we need HEV vaccines specific to particular genotypes for immunization, or can HEV vaccines confer cross-genotype protection? This question is of vital importance.

All HEV variants infecting humans share a single distinct serotype, demonstrating substantial immunological overlap and cross-reactive immunity across both primate models and human clinical subjects [132,133,134]. Consequently, recombinant HEV-239, although genotype-specific, is expected to confer broad protection across all four HEV-gts [104]. In the phase III trial, the vaccine was derived from an HEV-gt1 strain, while HEV-gt4 was the predominant genotype circulating in the study population in Dongtai County. The study recorded 23 HEV cases: 22 in the control group and 1 in the vaccine group. HEV-gt identification was successful for thirteen patients. Among these isolates (all in the placebo group), twelve were identified as HEV-gt4, while one was HEV-gt1 [113,135]. Thus, the HEV 239 -induced protection was primarily against HEV-gt4. Such cross-genotype efficacy remains possible because humans host only a single identifiable serotype of the virus [136,137]. Furthermore, structural research and animal challenge trials using HEV-gt1 identified antibodies that target rat HEV, suggesting potential prophylactic protection against rat HEV [132].

The committee is of the opinion that HEV 239 (Hecolin) showed efficacy against unrelated HEV-gt4 (prevalent in China). However, the efficacy of HEV 239 (Hecolin) against homologous HEV-gt1 (prevalent in India) and other HEV genotypes, such as HEV-gt3 (prevalent in the West), has not been quantified.

**Statement 9.** The hepatitis E vaccine should be administered to individuals regardless of antibody levels acquired through previous natural infection.

Consensus: evidence, moderate; benefit, moderate; recommendation, C.

Globally, the seroprevalence of hepatitis E in healthy populations varies but indicates significant past exposure, especially in Asia and Africa, with rates of around 12–21% for past infection (IgG-positive). At the same time, India shows variable rates (e.g., 2.75% to over 20% across different studies) depending on location and demographics, with a higher prevalence among older adults, and is of significant concern among pregnant women. [37,138,139]. During HEV immunization, a variable percentage of the population will be IgG anti-HEV-positive. Should we exclude subjects with natural immunity (as determined by prior IgG anti-HEV testing) or vaccinate them to achieve hybrid immunity?

To address this question, it is worth evaluating natural immunity, vaccine-induced immunity, and hybrid immunity (natural immunity plus vaccine-induced immunity) (Table 8) [140]. Natural immunity (in seropositive individuals) develops after exposure to varying doses of the virus [141]. The antibody levels vary widely with the severity of the initial illness and may be lower following asymptomatic low-dose exposure. The antibody level wanes over time, and approximately 50% of affected individuals become seronegative at 14.5 years [142,143]. Protection is partial (only against severe clinical disease), and subclinical infections continue to occur; virus transmission is not prevented [144]. Vaccine-induced immunity results in high, consistent, long-lasting antibody levels, and modeling predicts that 82–99% of individuals remain seropositive for more than 30 years after a full course of vaccination. It offers full (100%) protection against clinical and subclinical disease and prevents the spread of the infection. Hybrid immunity (in vaccinated seropositive individuals) elicits the highest antibody titers, significantly higher than those elicited by either natural or vaccine-induced immunity, and the antibody response is more durable and highly efficacious [145].

Thus, natural immunity has a variable antibody response, is short-lasting, and is partially protective. Testing and excluding such individuals from immunization is not advisable. Vaccination in such patients elicits the highest antibody response, which is more durable and highly efficacious and therefore recommended. Further, testing and vaccinating only seronegative individuals would make vaccination impractical, introduce delays, affect patient compliance, and may not be cost-effective.

Section B: The clinical indications of the HEV 239 (Hecolin^®^) in India.

**Figure 3:**
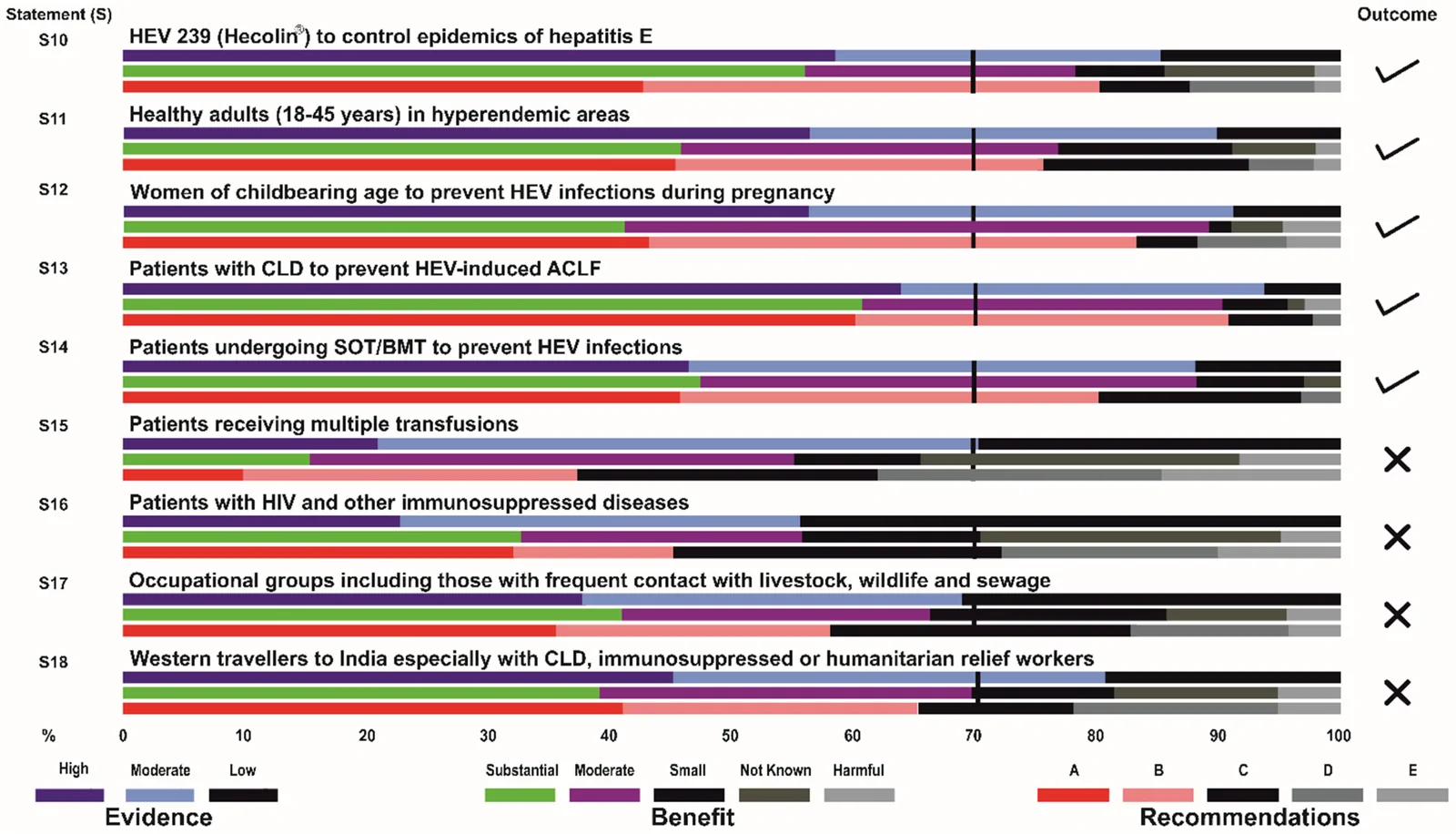
The cumulative response of the expert panel to the nine statements in section B. The responses were based on the GRADE system. We set 70% as the consensus threshold for accepting evidence, benefits, and recommendations (vertical bars). A positive report on the vaccine had to include a level of evidence of high or moderate, a potential benefit of substantial or moderate, and recommendations of A or B. Statements 10 to 14 were accepted (marked ✓ under Outcome), while statements 15 to 18 were rejected (marked ✕ under Outcome).

Nine statements in section B (items 10–18) were evaluated by the expert panel for their level of evidence, potential benefits, and recommendations. The cumulative three responses of the expert panel to each statement are shown in Figure 3.

**Statement 10.** A two-dose or accelerated three-dose schedule for HEV 239 (Hecolin^®^) is indicated for controlling hepatitis E epidemics.

Consensus: evidence, moderate; benefit, moderate; recommendation, B.

Hepatitis E epidemics are a common occurrence in India due to unsafe water supplies and poor sanitation, as seen following the monsoon season, due to sewage-contaminated public water supplies, fecally contaminated open water supplies, etc. Epidemics have been reported in vulnerable areas like refugee camps, overcrowded localities, slums, and military installations. These epidemics cause death and devastation, raising public concern and fear [44,72,146,147]. During the years 2013 to 2018, the Integrated Disease Surveillance Program (IDSP) reported 78 epidemics of hepatitis E in India, with 22,671 cases and 152 deaths [46]. Given the common source, short duration, and explosive nature of the epidemic, measures to control it are inadequate and ineffective.

Hepatitis E outbreaks manifest in several epidemiological patterns. The majority of hepatitis E epidemics are caused by contamination of the drinking water with sewage. These outbreaks are often “explosive,” unimodal, and characterized by a highly compressed epidemic curve, with cases appearing over a relatively short period of a few weeks. Secondary waves of hepatitis infection usually do not occur following epidemics, suggesting that person-to-person contact transmission is not a major factor in the evolution of such outbreaks [8]. A vaccination program to control short, massive epidemics requires a targeted preemptive vaccination strategy combined with rapid-response protocols. Since the disease is explosive and short-lived, reactive mass vaccination during an ongoing outbreak is often impractical due to the time required to build immunity. A targeted high-risk group for vaccination includes those with CLD, SOT, and hematopoietic neoplasms, PLWH, and other immunocompromised individuals. Vaccinating pregnant women would require the availability of safety data and approval from the relevant authorities [114].

After the first wave, an epidemic shows one or more peaks due to continued contamination of water sources. Epidemics reported from Kashmir presented as multiple waves of water-borne common-source epidemics affecting North Kashmir, South Kashmir, and Jammu [148]. A vaccination program would prevent such peaks. Some hepatitis E outbreaks are protracted, lasting months to years. These often occur in crowded, confined settings, such as urban slums and camps for internally displaced people (IDPs). The transmission may be multifactorial, involving contaminated drinking water supplies, person-to-person transmission, and practices such as open defecation and hand-to-mouth washing in a common family bowl before and after each meal [44,149]. A vaccination program to control such epidemics, using an accelerated dosing schedule, would be most effective.

The HEV239 (Hecolin^®^) was administered in a two-dose schedule in adults (aged 16–40 years, including pregnant women) during an outbreak of hepatitis E in Bentiu IDP camp, South Sudan, and Fangak County. The vaccine effectiveness was 89.4% [130,150,151].

The committee believes that hepatitis E epidemics constitute a significant health risk in India. At present, measures to control such outbreaks are inadequate and ineffective. The vaccine, on an accelerated or two-dose schedule, should be used to control such epidemics and to gain long-term data.

**Statement 11.** Hepatitis E vaccination should be offered to healthy adults (18–45 years) in hyperendemic areas to prevent sporadic HEV infections and acute liver failure.

Consensus: evidence: moderate; benefit, moderate; recommendation, B.

HEV is a leading, often the most common, cause of acute viral hepatitis and acute liver failure (ALF) in India, particularly among young adults and pregnant women. It is usually spread via contaminated water. An estimated attack rate of 0.5–1.2 per 1000 per year and a seroprevalence of 27.15% define the magnitude of hepatitis E in India. While hepatitis A (HAV) is also common, especially in children, HEV is a major driver for severe hepatitis and ALF cases across the country. Recent short-term data have shown changes in the epidemiology of HAV and HEV in India: the prevalence of HAV is higher than that of HEV, and HAV and HEV peak among children and adults, respectively, during the monsoon season. These data may be related to several factors, including the cohort phenomenon (HEV), improved water supplies and sanitation, and the introduction of more aggressive HAV in the community. The committee believes that hepatitis E continues to be a significant cause of morbidity and mortality in India. A large-scale phase III trial in China demonstrated the safety, immunogenicity, and long-term effectiveness of the HEV vaccine p239 (Hecolin^®^).

**Statement 12.** The hepatitis E vaccine should be offered to women of childbearing age to safeguard them from hepatitis E-related morbidity and mortality during pregnancy.

Consensus: evidence, moderate; benefit, moderate; recommendation, B.

**Figure 4:**
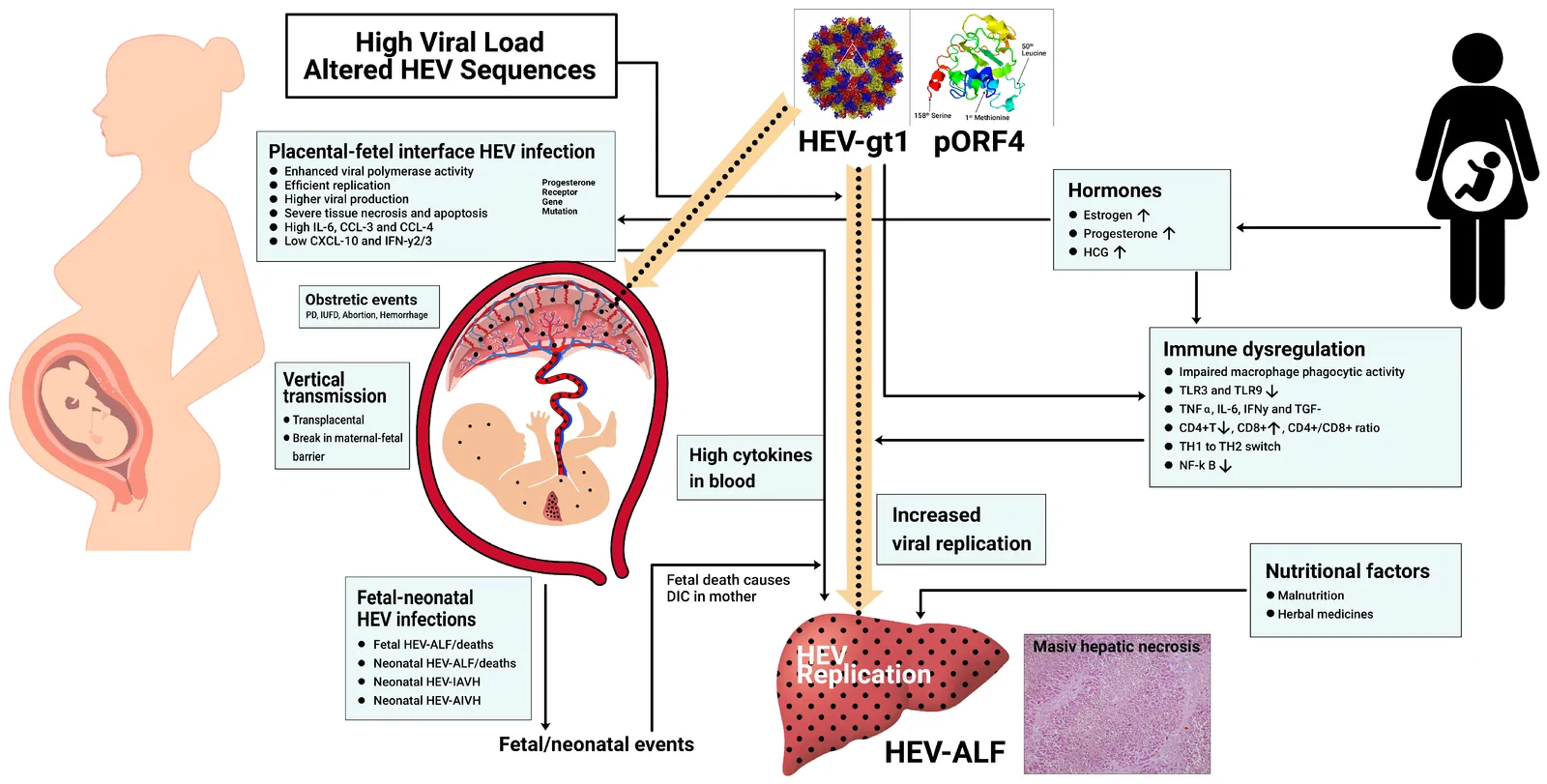
Proposed pathogenesis of the adverse relationship between hepatitis E and pregnancy. [See text for details. HEV-ALF = hepatitis E virus-related acute liver failure. IAVH = Icteric acute viral hepatitis. Adopted from [13].

Hepatitis E is significantly more severe during pregnancy. Pregnant women face exceptionally high rates of fulminant liver failure, maternal mortality, and poor obstetric outcomes due to HEV infection, especially in the third trimester (Table 9) [52,152,153,154] The pathogenesis of HEV-induced severe maternal disease is complex, poorly understood, and unique to HEV-gt1 alone. It is caused by hormonal immune dysregulation, increased viral replication, and HEV infection of the placental–fetal interface and the fetus. Pregnancy hormones are known to shift the immune response from a Th1-dominant (pro-inflammatory) state to a Th2-dominant (anti-inflammatory and tolerogenic) state and to promote the proliferation and activity of anti-inflammatory regulatory T cells (Tregs) [52]. This adaptation protects the developing fetus, but it increases immune-mediated viral replication. HEV-gt1 has an additional ORF4 in its genome, which encodes a small 120-aa pORF4. pORF4 is expressed from an alternative reading frame and is regulated via an IRES-like RNA element. It is indispensable for the life cycle of HEV-gt1 and increases viral replication. It also promotes HEV infection at the placental–fetal interface, leading to tissue necrosis, apoptosis, vertical transmission, and cytokine release into the maternal circulation. Fetal HEV infection often leads to massive hepatic necrosis and fetal death, which can cause DIC in the mother (Figure 4) [153,155].

Hepatitis E vaccination is strongly indicated in this high-risk population. While clinical data generally indicate that the vaccine is safe and well-tolerated when administered during pregnancy, no global regulatory agencies have officially approved its use for pregnant women at this time [116,156,157]. There are several reasons for this. First, pregnant women are excluded from early vaccine trials, and evidence primarily relies on studies of women who inadvertently received doses early in their pregnancy [115,157]. Studies from Bangladesh have revealed a potential increased risk of miscarriage when administered in the 90 days before conception or early in the first trimester [115]. The WHO does not recommend routine vaccination for pregnant women outside of outbreak settings [121]. The authorities in India have recommended hepatitis E vaccination for pregnant women strictly before conception [27].

In view of the above, hepatitis E vaccination in women of childbearing age is an alternative. The vaccine triggers a strong, long-lasting immune response, effectively blocking the virus from attaching to and entering liver cells [115]. A phase IV, double-blind trial on the safety and effectiveness of HEV 239 in women of childbearing age was conducted in Bangladesh. Of the 19,460 participants, 8524 received three doses of the HEV-239 vaccine, compared with 9089 who received the hepatitis B vaccine. The vaccine was safe, with no SAEs. Seroconversion occurred in 97.7% of the vaccine group versus 10.3% in the control group. Among pregnant participants, the vaccine group had a higher risk of miscarriage. No case of hepatitis E occurred in pregnant women in either group, and the effectiveness of the vaccine in pregnant women remained uncertain [115]. Vaccinating women before they become pregnant ensures they already have robust, circulating antibodies, which will prevent maternal infection and the subsequent risks of vertical transmission, spontaneous abortion, or stillbirth. However, there are no data available on the impact that vaccinating women of childbearing age would have on the outcome of hepatitis E in pregnant women in India.

**Table 9:** Relationship of hepatitis E with pregnancy in India.

	References
**Epidemic HEV-AVH and pregnancy**	[54]
During epidemics, pregnant women acquire HEV infection 8 times more than non-pregnant women and men (15–45 years): 8.8%, 19.4%, and 18.6% in the three trimesters.	
Around one-fourth (22.2%) of HEV-infected pregnant women develop ALF compared with 1.6% in HEV-infected non-pregnant women and men (15–45 years), with rates of 0%, 0%, and 44.4% in the three trimesters, respectively.	
CFR among pregnant women is 16.6% compared with 1.67% in non-pregnant women and men (15–45 years) and is 0%, 0%, and 33.3% in the three trimesters, respectively.	
**Sporadic HEV-AVH and pregnancy**	[51,53]
HEV infection is the cause of sporadic AVH in around 85.5% of pregnant women as against 41.5% in non-pregnant women and men (15–45 years).	
ALF develops in around 69.2% of pregnant women with HEV infection as against 10.0% in non-pregnant women and men (15–45 years).	
**HEV-ALF and pregnancy**	[49]
Around 95.8% of ALFs in pregnant women are caused by HEV as against 41.1% in non-pregnant women (15–45 years).	
HEV-ALF in pregnant women is an explosive disease with short PEP, high occurrence of cerebral edema, and DIC.	
CFR in HEV-ALF is 51.9% and is significantly less than CFR in non-HEV-ALF (84.2%). Pregnancy per se or duration of pregnancy did not adversely affect prognosis	
**Obstetric complications in pregnancy with HEV infection**	[54,158,159]
Pregnant women with HEV infection had a higher occurrence of obstetric complications than those with non-HEV infection.	
Obstetric complications in HEV-infected pregnant women include preterm labor, antepartum hemorrhage, intrauterine fetal deaths, abortions, and neonatal deaths	
**Vertical transmission of HEV and its implications**	[153,155,160]
Vertical (transplacental) fetal/neonatal HEV infection is reported to occur in 33–100% of pregnant women with HEV infection.	
About 15% of HEV-infected fetuses either die in utero or abort. Liver histology shows massive hepatic necrosis.	
Around half of the HEV-infected neonates develop ALF and present with hypoglycemia, hypothermia, and death. Of the remaining, around one-fourth develop self-limiting acute hepatitis (30.4%) or anicteric hepatitis (22%) without chronic viremia, hepatitis, or chronic liver disease.	
Mothers who deliver HEV-infected babies early (within 4 days of disease) survive more than those who deliver late. DIC in mothers with HEV-ALF occurred exclusively when the fetus/neonate had an HEV infection with massive hepatic necrosis.	

Adopted from: Khuroo MS. Discovery of Hepatitis E and Its Impact on Global Health: A Journey of 44 Years about an Incredible Human-Interest Story. Viruses. 2023 Aug 15;15(8):1745. doi: 10.3390/v15081745. PMID: 37632090; PMCID: PMC10459142. [13] ALF = Acute liver failure; AVH = acute viral hepatitis; CFR = case fatality rate; DIC = disseminated intravascular coagulation.

**Statement 13.** Patients with chronic liver disease, including those with cirrhosis and chronic HBV, should receive the hepatitis E vaccine to prevent HEV-induced Acute-on-Chronic-Liver-Failure (HEV-ACLF).

Consensus: evidence, moderate; benefit, moderate; recommendation, B.

ACLF is a life-threatening syndrome in which a person with pre-existing CLD suddenly develops acute liver decompensation and multiorgan failure. Driven by an intense systemic inflammatory response (“cytokine storm”), this condition carries a very high short-term mortality rate exceeding 20% within 28 days if left untreated. Unlike standard end-stage liver disease, ACLF is characterized by its abrupt onset and potential reversibility if the primary trigger is quickly managed [61]. HEV is also a common cause of superinfection precipitating ACLF in patients with underlying CLD due to various etiologies [161]. HEV RNA was detected in 28% of patients with cirrhosis, leading to the conclusion that cirrhotic patients are particularly prone to HEV infection, which is associated with rapid hepatic decompensation and increased mortality [57,58,59].

Vaccination against HEV in patients with CLD, cirrhosis, and chronic hepatitis B virus (HBV) infection represents an attractive preventive strategy to reduce the incidence of ACLF and associated mortality. A post hoc analysis evaluating the immunogenicity and safety of the HEV-239 vaccine in HBsAg-positive CLD patients demonstrated immune responses comparable to those observed in HBsAg-negative individuals [109]. Furthermore, a recent clinical trial provided robust evidence supporting the safety and immunogenicity of the HEV vaccine in patients with chronic hepatitis B and compensated liver cirrhosis [122]. In this study, all cirrhotic participants seroconverted 1 month after completing the vaccination schedule, with 82.8% achieving high anti-HEV IgG titers (>1 WU/mL), and no significant adverse events were reported.

Collectively, these findings strongly support the use of the HEV vaccine as a safe and effective intervention to prevent HEV superinfection in patients with underlying CLD. Consequently, the European Society of Clinical Microbiology and Infectious Diseases Viral Hepatitis Study Group recommends universal HEV vaccination for patients with CLD, irrespective of circulating genotype, given the substantial risk of HEV-induced ACLF [25]. The committee advocates immunization in patients with CLD of all etiologies.

**Statement 14.** Patients undergoing solid-organ or bone marrow transplants should receive the hepatitis E vaccine to guard against HEV infection.

Consensus: evidence, moderate; benefit, moderate; recommendation, B.

Advice on the use of hepatitis E vaccination in SOT recipients and other immunocompromised patients is highly logical but clinically complex. The accumulated data on hepatitis E in SOT and other immunocompromised patients are from developed countries, where HEV-gt3 is prevalent [2]. HEV-gt3 is known to cause chronic hepatitis and cirrhosis in such patients [23]. In fact, chronic hepatitis E is a major health problem in SOT recipients, patients with hematopoietic neoplasms, and PLWH in the West [162,163]. The burden of disease in SOT recipients is high (around 20%), with liver transplant patients at the highest risk (27.2%), followed by renal/heart transplant (12.8%) and lung transplant (5.6%) patients [164]. These infections can cause chronic hepatitis and cirrhosis in vulnerable hosts. Current protocols for treating acute cases focus on adjusting immunosuppressive regimens, particularly by removing calcineurin inhibitors. Supplementary ribavirin treatment has demonstrated favorable outcomes in viral clearance and is a standard clinical protocol. Nevertheless, prophylactic immunization before the transplant might avert chronic infection. Studies in immunocompromised rabbit models of chronic HEV confirm that administering the vaccine *prior* to immunosuppression confers complete protection against both HEV-gt3 and HEV-gt4. However, if vaccination is administered during ongoing immunosuppression, protection is only partial, even with increased or additional doses [165]. These findings indicate that SOT candidates should receive the HEV vaccine before starting immunosuppressants to ensure optimal therapeutic efficacy.

In India, hepatitis E is driven by HEV-gt1. HEV-gt1 is not known to cause chronic liver disease in either healthy populations or in SOT recipients [13]. However, SOT recipients in India have a higher IgG anti-HEV seroprevalence and elevated liver test values [166,167]. Although it has not been fully explored, acute HEV superinfection among unvaccinated SOT recipients in India can still cause severe hepatitis due to their compromised immune status. Therefore, advice on hepatitis E vaccination for SOT recipients in India seems medically sound but should be considered with realistic expectations. The aim of vaccinating SOT recipients in India is to prevent acute symptomatic infection rather than avert the chronic, progressive liver fibrosis seen with HEV-gt3. The magnitude of acute hepatitis E in such patients requires further study, and future real-world data are required to determine the impact of vaccinating such patients.

**Statement 15.** Patients who require multiple blood transfusions or blood products should receive the hepatitis E vaccine.

Consensus: evidence: moderate; benefit, not known; recommendation, D.

In industrialized countries, transfusion-transmitted hepatitis E (TT-HEV), caused by HEV-gt3 and HEV-gt4, is recognized as a significant emerging threat. A variable proportion (0.001% to 0.28%) of healthy blood donors are viremic and can transmit HEV [168]. In 2013, an estimated 1200 HEV-containing blood components were transfused in England. Follow-up of 43 such recipients showed that 18 had evidence of HEV infection [168]. Based on a 0.008% prevalence of HEV RNA in donor samples and 7.4 million blood products donated, it was estimated that between 1600 and 5900 HEV-RNA-positive blood donations occurred in Germany in 2013 [169]. Such infections can cause chronic hepatitis and cirrhosis in vulnerable immunosuppressed individuals [170]. A meta-analysis of Chinese blood donors reported substantial prevalence rates for HEV IgG (29.2%), HEV IgM (1.1%), HEV RNA (0.1%), and HEV antigen (0.1%) [171]. Recognizing the burden of transfusion-transmitted hepatitis E, several countries, including the UK, Ireland, Japan, Germany, and Switzerland, have implemented mandatory or universal screening of blood donations for HEV RNA using nucleic acid testing (NAT) [172,173]. From 2004 to 2020, 45 cases of transfusion-transmitted hepatitis E were reported in Japan. Universal NAT was implemented in August 2020. A total of 50,075,100 blood donations were screened in the first year. HVV RNA (detection limit > 205 IU/mL) was detected in 2804 (0.055%) samples, potentially causing an estimated 270,000 new HEV infections in Japan annually. Among the 1113 cases in which the HEV genotype was determined, HEV-gt3 was detected in 1100 (98.8%) samples and HEV-gt4 in 13 (1.2%). In the three years since NAT was implemented, no case of TT-HEV has been detected [174,175].

Transfusion-transmitted HEV infections caused by HEV-gt1 have been reported in India in prospective and case–control studies [176]. Three clinical HEV infections were traced to HEV RNA-positive donor samples. In a study from Pune, Maharashtra, India, the HEV prevalence was 17.70%, with 5 of 2447 (0.20%) healthy donors positive for anti-HEV IgM and 2 positive for HEV RNA (138). In another study from Surat, India, based on pooled HEV RNA testing, 7 (0.53%) of 130,500 donations were HEV RNA-positive, and the prevalence of HEV RNA among blood donors was 1 in 1864 [177]. These studies indicate that there is a significant risk of transfusion-transmitted HEV infections in India [178]. However, a follow-up of 2000 transfused patients failed to detect any cases of HEV infection [179].

There are no universal, society-wide guidelines recommending the routine use of the hepatitis E vaccine for all multiply transfused patients. However, there is a need for vaccinating multiply transfused patients, namely those with thalassemia and sickle cell anemia. Transfusion-transmitted HEV infection in pregnant women and patients with CLD in India can progress rapidly to severe clinical disease. Protecting such groups prior to transfusion is worth considering.

**Statement 16.** Patients with PLWH and those with other immunosuppressed states should receive the hepatitis E vaccine.

Consensus: evidence, low; benefit, small; recommendation, C.

The prevalence of HEV exposure among PLWH has been reported to be as high as 40% in Africa and Asia. HIV-induced immunosuppression predisposes patients to chronic HEV infection [24,180]. Therefore, HEV vaccination may be considered as a preventive strategy to reduce the risk of HEV infection and its complications. Patients on maintenance hemodialysis undergo frequent invasive medical procedures and often receive blood products. Parenteral transmission of HEV alongside HBV and HCV is a potential risk among such patients. In one study, 42 out of 405 hemodialysis patients were positive for anti-HEV IgG (10.4%) [181]. In another study from Ankara, Turkey, a higher seropositivity rate of 26.25% was observed among hemodialysis patients, while other cohorts have reported rates as low as 3% [182]. The safety and immunogenicity of the hepatitis E vaccine have been evaluated in patients undergoing maintenance hemodialysis. The overall incidence of adverse reactions or events was 17.1% (18/105), with no vaccine-related grade 3 or 4 adverse events reported. Among the 23 patients who received three doses according to the standard vaccination schedule, the HEV-IgG seropositivity rate was 100%, with a geometric mean concentration (GMC) of 14.47 WU/mL (95% CI: 13.14–15.80). This immune response was comparable to that observed in 46 participants from the Phase III clinical trial, with no statistically significant difference (t = 1.04, *p* > 0.05). These findings indicate that the recombinant HEV vaccine is safe and immunogenic in patients undergoing maintenance hemodialysis [183].

Data on the use of the hepatitis E vaccine in PLWH and other immunosuppressed populations are evolving. HEV 239 is a recombinant protein, not a live or attenuated virus, and should therefore be safe in this population. While the vaccine may be safe for use, the magnitude and durability of the antibody response in immunosuppressed patients may be lower than in immunocompetent individuals, and tailored dosing or additional booster shots may be needed [184].

**Statement 17**. Occupational groups, including workers who have frequent contact with livestock, wildlife, and sewage, should receive the hepatitis E vaccine.

Consensus: evidence, low; benefit, small; recommendation, C.

People with several occupations, specifically veterinarians, swine industry workers, farmers, and hunters, contend with a heightened susceptibility to HEV due to their frequent and direct interaction with natural animal reservoirs [81]. Similarly, military personnel may be at increased risk of HEV infection as a result of vulnerable living conditions during field deployment or travel to HEV-endemic regions. Military environments often involve shared facilities and potential exposure to contaminated food or water, thereby elevating the risk of HEV transmission [185,186].

The hepatitis E vaccine represents an important preventive measure with the potential to significantly reduce the risk of severe illness and minimize operational disruption in these high-risk groups. Although no clinical trials have specifically evaluated the efficacy of HEV vaccination in preventing infection among these occupational populations, the substantial risk of exposure supports consideration of vaccination.

**Statement 18.** Western travelers to India, especially those with CLD, immunosuppressed individuals, or humanitarian relief workers responding to an outbreak, should receive the hepatitis E vaccine.

Consensus: evidence, moderate; benefit, moderate; recommendation, C.

Hepatitis E is endemic in India and typically spreads through water contaminated with sewage. For most tourists, the primary prevention strategy is to pay close attention to food and water safety. The primary recommended vaccines for travelers to India to prevent food- and waterborne diseases are Hepatitis A and Typhoid. However, acquisition of hepatitis E infection in Western tourists cannot be ruled out [187]. A multicenter retrospective study of HEV in travelers to developing countries between 1999 and 2018 revealed 142 cases of HEV infection. HEV infections occurred in 41% of cases during travel to India, and 56% of patients had clinical disease requiring hospitalization. Only one pregnant woman developed an HEV infection, and no deaths were reported [188]. Shlim et al., who support the use of the hepatitis E vaccine in travelers to endemic areas, believe that most HEV infections in travelers go unreported [189]. Given the risk to travelers, hepatitis E vaccination has been recommended for those traveling to endemic areas [190].

While the HEV 239 has been approved and launched in India for local use in adults aged 18 to 65 years, it is not included in the standard vaccination schedule for international travelers. Vaccination may be considered on an individual basis for travelers at higher risk of severe disease, such as those with pre-existing chronic liver disease or immunosuppression, or for humanitarian relief workers responding to an outbreak.

**Figure 5:**
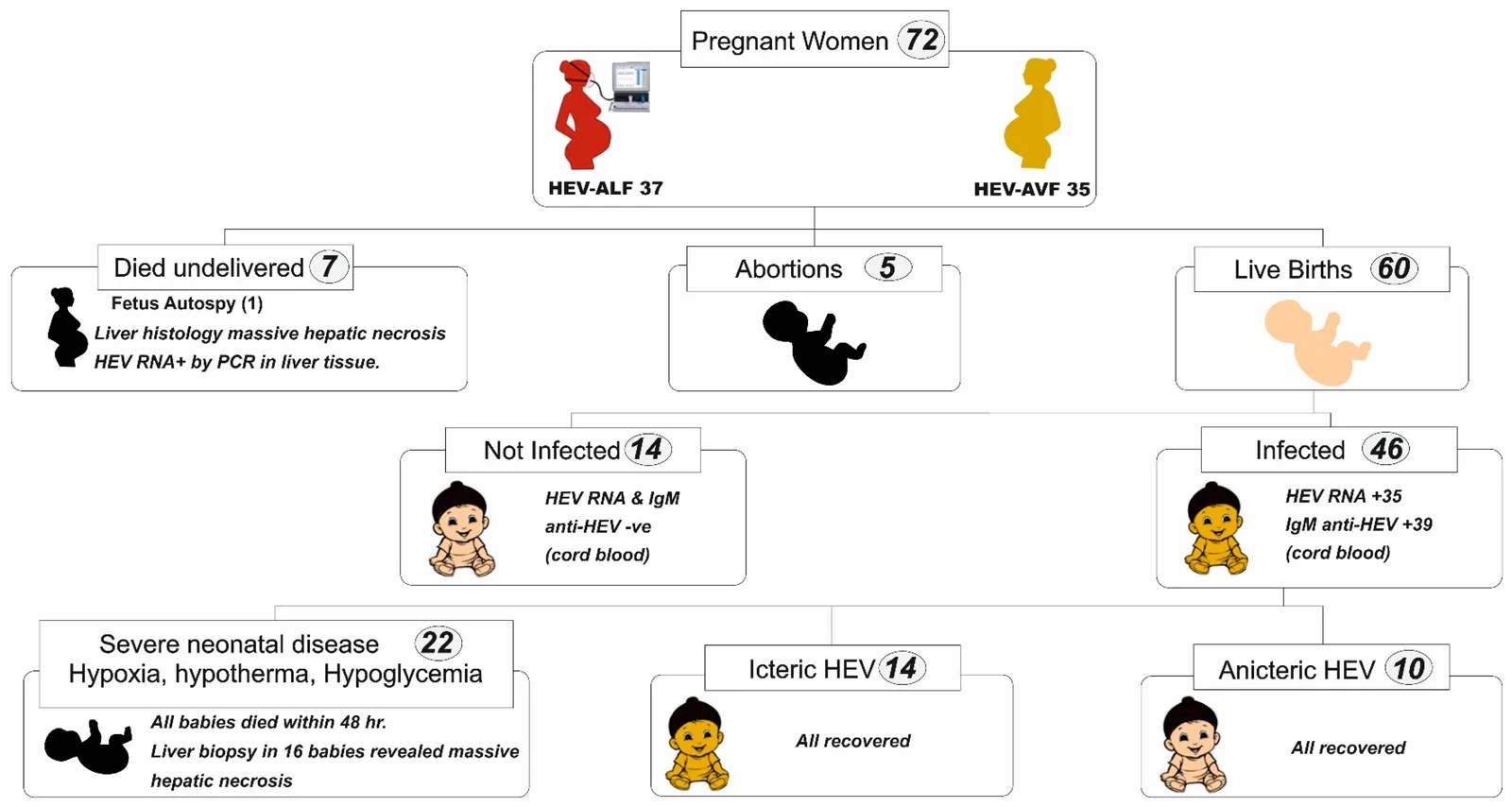
Hepatitis E infection in fetuses and neonates of 72 pregnant women with HEV-gt1 infection at SKIMS from 1993 to December 2022. Of the 72 pregnant women with HEV infection, seven fetuses died undelivered, five aborted, and 22 newborns died within 48 h of birth with HEV-related fulminant hepatic failure; 14 neonates developed self-limiting icteric HEV infection, and 10 had anicteric HEV infection. Newborn babies with HEV infection had prolonged viremia, in a few cases lasting up to 32 weeks. However, chronic hepatitis or cirrhosis did not occur in any of the babies (ALF = acute liver failure) [153,155,160].

**Neonatal hepatitis E.** Neonatal HEV infection is a significant health problem in developing countries. (Figure 5) The currently licensed HEV 239 (Hecolin) is formulated and approved only for adults. HEV infection in neonates is almost exclusively caused by vertical transmission from an infected mother; clinical management must address both the infant and the source of the infection. [52,152,153]. Vaccinating women of childbearing age to prevent HEV infection in pregnant women would protect both the mother and the neonate from adverse effects.

**HEV infections in Adolescents**: Clinical trials of the hepatitis E vaccine have been conducted in adults aged 16 to 65 years. At present, no trials of the vaccine have been conducted in children. Clinical disease with HEV in India occurs predominantly among young adults aged 15 to 45 [191]. In most healthy children, HEV infection is either asymptomatic or causes a very mild, self-limiting illness. Severe or fatal complications have been reported in children, but they are quite rare. Why young children are spared from infection transmitted through the feco-oral route remains unknown. In Egypt, the epidemiology of HEV resembles that of HAV, and children demonstrate a high seroprevalence of HEV [192]. We believe the childhood population is not a primary focus for hepatitis E vaccination. Despite this, the lack of pediatric data on the hepatitis E vaccine is problematic for several reasons. First, specific groups of children require protection against HEV infection. These include children with compromised immune systems, such as SOT recipients, patients with hematopoietic neoplasms, PLWH, and children suffering from CLD. Clinical trials initiated and underway in Pakistan and Sudan may address the safety and efficacy of hepatitis E vaccination in children.

**Consensus.** We plotted the 33 cumulative responses to 18 statements in bar charts, as shown in Figure 1 and Figure 3. As previously mentioned, we set 70% as the consensus threshold for accepting evidence, benefits, and recommendations. Of the statements (1 to 9) related to the safety, immunogenicity, and efficacy of the HEV-239 vaccine, seven statements reached the acceptance threshold. Statements related to cross-genotype protection (statement 8) and the benefit of vaccinating subjects irrespective of their prior antibody status (statement 9) did not cross the 70% threshold.

The safety, immunogenicity, and efficacy of the HEV-239 vaccine have been confirmed. Consensus was reached at the 70% threshold for statements 10–14. Based on this, we recommend a targeted vaccination strategy across five clinical scenarios in India. These include the following:

1. Vaccinate high-risk groups with a two-dose or accelerated three-dose schedule during outbreaks or epidemics of hepatitis E.
2. Vaccinate high-risk groups in highly endemic pockets of hepatitis E in India.
3. Vaccinate women of childbearing age in endemic areas to target the occurrence of hepatitis E during pregnancy.
4. Vaccinate patients with CLD of any etiology to prevent the occurrence of HEV-ACLF.
5. Vaccinate SOT recipients prior to transplantation and immunosuppression.

It is advisable to maintain records of such vaccinations, including adverse effects, the subjects’ immune responses (if studied), and long-term outcomes. This will aid in formulating a hepatitis E vaccination policy and strategy in India.

The cumulative responses of the experts did not reach the 70% threshold for vaccinating subjects who require multiple transfusions (statement 15), PLWH and other immunosuppressed (statement 16), subjects who work with livestock (statement 17), or Western travelers to India (Statement 18). The main reason was that the data available for these groups were not robust enough to reach the consensus threshold. The steering committee believes that vaccinating these groups would be beneficial and recommends doing so to generate real-world outcome data.

## 4. Discussion

The consensus statements in this study were based on an in-depth literature search on the epidemiology of hepatitis E in India, as well as data on the safety, immunogenicity, and effectiveness of HEV 239. The consensus-based recommendations have inherent advantages and limitations. The statements were carefully synthesized to highlight potential uses of the vaccine in clinical practice. Thirty-three highly distinguished and experienced Indian hepatologists graded the levels of evidence, potential benefits, and recommendations. The level of evidence was based on the thoroughness of the literature review. The opportunity for unrestricted individual feedback and discussion between the experts and the steering committee allowed for independent thinking without creating confrontation or allowing dominant voices to skew the process. The experts often submitted queries and requested full-text articles of seminal publications. The experts’ feedback was incorporated into the statements. The potential benefits were evaluated based on the balance between desirable and undesirable effects and required the expert’s experience. The grades of recommendation required expert judgment to make a clinical decision based on all available data and overall healthcare needs. We used a modified Delphi process and maintained anonymity to eliminate dominating opinions, avoid peer pressure, and ensure equal weight [193]. The use of the ACCORD (Accurate Consensus Reporting Document) guidelines ensures transparency, eliminates groupthink, and produces scientifically rigorous reporting. It harnesses collective expert intelligence through iterative, anonymous voting, structured feedback, and critical literature reviews [194]. However, we encountered several scenarios in which the evidence was either not robust or unavailable. In such situations, statements were based on reviews from apex committees including WHO, SAGE, the European Society of Clinical Microbiology and Infectious Diseases (ESCMID), and the International Vaccine Institute (IVI). In addition, we listened to the opinion of our highly respected expert panel. We believe these do not allow personal bias to influence the consensus. Also, consensus opinion fundamentally depends on team members’ efficiency, which is poorly reproducible [195].

As the HEV-239 (Hecolin^®^) is proposed for use in India but lacks local clinical data, consensus is the most critical bridge between foreign trial data and local implementation. Experts evaluate whether clinical trial findings from China are biologically applicable to the Indian population and determine whether genetic and environmental variations will alter vaccine efficacy. Consensus mitigates the inherent risks of introducing a novel foreign drug by applying collective scientific judgment to assess clinical efficacy and ensure it meets domestic safety standards [196].

The opinions of multiple international agencies and research groups indicate that the HEV-239 vaccine is highly efficacious and provides long-term protection, yet its widespread adoption is hindered by significant regulatory, logistical, and data gaps [25,184,197,198,199,200,201,202,203] While the World Health Organization (WHO) initially declined to recommend its routine use in 2015 due to insufficient data on children, pregnant women, and cross-genotype efficacy, more recent 2024 SAGE updates conclude that in fragile and conflict-affected settings, the benefits of vaccinating women of childbearing age outweigh potential harms [198,199]. Major barriers identified by the IVI and the ESCMID include a lack of WHO prequalification, which restricts global procurement and funding from bodies such as Gavi, as well as cold-chain requirements and limited awareness among policymakers [25,197,200]. Despite a concerning safety signal regarding spontaneous abortion in a Bangladesh trial, which researchers emphasize requires further investigation, real-world data from outbreak responses in South Sudan have shown the vaccine to be safe and effective, leading to new global policy initiatives to establish a vaccine stockpile for emergency responses [115,116]. Following these developments, European groups now universally recommend HEV vaccination for high-risk groups, such as those with chronic liver disease and SOT recipients, while calling for clinical trials to assess the vaccine’s specific impact within Europe [25,26].

Most trials of the HEV 239 have been conducted in China. While the trial data demonstrate that the vaccine is highly safe and efficacious, they limit the universal applicability of the results in several ways [203]. First, studies raise the issue of HEV-gt mismatch. The HEV-239 vaccine was produced from HEV-gt1 and has been shown to provide cross-protection against the prevalent HEV-gt4 [113]. Its direct efficacy against homologous HEV-gt1 remains unquantified. Moreover, its efficacy may vary against HEV-gt2 and HEV-gt3, which are prevalent in other regions of the world [2]. Second, most Chinese trials have been conducted in healthy adults aged 16 to 65 years. There are limited or no conclusive clinical data on the vaccine’s efficacy and safety for high-risk groups like pregnant women, immunocompromised individuals, SOT recipients, and children under 16 years of age. Third, trials were conducted in an environment with distinct dietary habits. These limit the generalizability of results across different populations, as background environment and diet profoundly influence baseline metabolic profiles, gut microbiomes, and overall health outcomes [204]. These facts emphasize the need for broader clinical research and post-marketing surveillance in other endemic and hyperendemic regions worldwide, including India.

Hepatitis E epidemics are a frequent occurrence in India and cause significant morbidity and mortality [205]. It is advisable to focus immunization efforts on the most vulnerable population before the epidemic season. Hepatitis E outbreaks are a frequent occurrence after the monsoon season, and a vaccination protocol for overcrowded localities and slums would be advantageous. Vaccination would be advisable in refugee camps or flood-displaced communities. India has a large interstate “Internally Displaced Population (IDP)” who work as daily laborers on makeshift sites with poor hygienic and sanitary conditions. Targeting this mobile population would be advisable to control the spread of hepatitis E [206].

Hepatitis E is a major health challenge in endemic regions, driven primarily by HEV-gt1 [36]. The emergence of approved recombinant vaccines signifies a critical advancement, providing a vital tool for safeguarding high-risk populations and preventing severe morbidity [121]. This consensus report on the use of the hepatitis E vaccine in India establishes a robust clinical and epidemiological framework that can be confidently adapted for use in other regions with high HEV-gt1 endemicity. These regions include countries adjacent to India, such as Bangladesh, Nepal, and Pakistan [207]. Other important regions include Sub-Saharan and North Africa, where hepatitis E frequently causes outbreaks in conflict zones, refugee camps, and areas with compromised sanitation, particularly in the Lake Chad region (e.g., Niger, Chad) [208,209,210,211]. HEV-gt1 is also endemic in Tajikistan and the surrounding areas, as well as in several countries in the Middle East [38,212].

Because HEV-gt1 dominates waterborne outbreaks in developing nations across Asia and Africa, the vaccine’s proven immunogenicity, safety, and high efficacy against homologous strains make it a transformative public health intervention [44,113]. By mitigating the threat of acute liver failure, the vaccine provides essential protection. While routine population-wide vaccination is not universally advised at this time, regional deployment should prioritize highly vulnerable demographics, including pregnant women, individuals with chronic liver disease, and populations exposed to a compromised water supply. The successful implementation of these guidelines in India will serve as a foundational, evidence-based blueprint that allows other highly endemic regions globally to curtail morbidity, protect vulnerable populations, and systematically manage hepatitis E outbreaks.

Managing vaccine hesitancy among doctors and the public regarding the hepatitis E vaccine remains a challenge [213]. Physicians are the most trusted source of vaccine information for patients, but hesitancy among doctors often stems from knowledge gaps about regional guidelines or vaccine safety. Through tailored medical education, we must provide clear clinical updates outlining which patients can benefit from hepatitis E vaccination [214,215]. These groups should include pregnant women, patients with CLD, SOT recipients, internally displaced populations, and travelers to HEV-endemic areas. Training doctors on the vaccine’s safety profile and duration of protection is important, and we need to encourage them to use available peer-reviewed studies to address questions about the vaccine’s recombinant technology. Public hesitancy is largely fueled by a lack of disease awareness (complacency) and concerns about vaccine safety [216]. To combat this hesitancy, we need to encourage community leaders, pharmacists, and patient advocacy groups to share accurate, culturally responsive information. A system to combat misinformation through Fact-First communication is required; vaccine myths should be addressed by explaining the risks of vaccination, as determined by clinical trials and real-world data. Information needs to be conveyed to the public through visual decision aids and simple infographics, which are particularly helpful for populations with lower health literacy. Institutional and public health authorities are essential components of vaccination and need up-to-date information on HEV vaccine procurement, availability, and specific regional recommendations. Vaccination sites should be accessible and operational, with transparent communication channels open to address any concerns about adverse events in real time.

No cost-effectiveness studies for the HEV 239 (Hecolin) have been conducted in India. A study from China found that universal preventive screening in a sporadic setting, combined with a three-dose vaccine schedule, was cost-effective only for patients with CLD and not for pregnant women or women of childbearing age. However, in an outbreak setting, a two-dose schedule would prevent 70% of deaths within six months and would be cost-effective for pregnant women, women of childbearing age, and the elderly [217]. Given the current high cost of the vaccine, its three-dose schedule, and the need to achieve coverage in the Indian population of 1.4 billion, universal vaccination would be economically prohibitive. However, targeted vaccination for high-risk groups may be cost-effective. These include women of childbearing age, individuals with CLD, and SOT recipients. Hepatitis E causes high mortality in pregnant women, especially in the third trimester, with a case fatality rate (CFR) between 25 and 30% [52]. Vaccinating pregnant women and women of childbearing age would reduce symptomatic cases by 70%, significantly impacting CFR [157]. Vaccinating patients with CLD would reduce HEV superinfection in 58.6% of cases, with a survival benefit of 57.5% [123]. The impact of the vaccine on SOT patients in India is debatable, as prevalent HEV-gt1 infection does not progress to chronic hepatitis and cirrhosis. However, given the cost involved in the procedure and follow-up, it would be advisable to vaccinate such patients to avoid episodes of acute hepatitis. During an epidemic, implementing an accelerated dose schedule and targeted vaccination in high-risk groups, mainly pregnant women, is likely cost-effective. This strategy showed an effectiveness of 89.4% (56.4 to 98.0) during an HEV outbreak in the Bentiu internally displaced persons camp, South Sudan [130]. Vaccinating only seronegative individuals would eliminate unnecessary vaccination costs and substantially reduce overall costs. However, this strategy may not be practical, as it raises issues of vaccination compliance.

This consensus report has several limitations. (Table 10) The main limitation is the lack of clinical data from the Indian population. Efficacy trials have been conducted in China, where HEV-gt4 is prevalent. The efficacy of this vaccine in the Indian population, among which HEV-gt1 is prevalent, has not been quantified. Additionally, differences in dietary habits and background environments between China and India may limit the generalizability of the existing trial results. The vaccine has shown some significant adverse effects in pregnant women from Bangladesh, which will hopefully be addressed in future trials. Vaccine safety, immunogenicity, and efficacy trials in patients with decompensated cirrhosis and other immunocompromised clinical states are not available. The expert panel failed to reach the 70% consensus threshold for several scenarios, such as vaccinating PLWH, multi-transfused individuals, or livestock workers, primarily because the available evidence was not robust enough. There is currently no cost-effectiveness study for the vaccine specifically conducted in India. Based on these facts, the current guidelines must serve as a baseline for vaccine use in India until real-world data can be collected to optimize future vaccination strategies.

## Figures and Tables

**Table 1:** Classification of the GRADE system (Grading of Recommendations Assessment, Development and Evaluation).

**LEVEL OF EVIDENCE**	
Synthesized from published data by the expert panel members.	
**High**	Consistent results from well-designed, well-conducted studies. The voidance is unlikely to be affected by future studies.
**Moderate**	Sufficient to determine the effects, but constrained by the number, size, or quality of individual studies; inconsistency of findings across individual studies; limited generalizability of findings to routine primary care practice; and lack of coherence in the chain of evidence. The magnitude or direction of the decisions could change, and such a change may be significant enough to alter the conclusions in future studies.
**Low**	The available evidence is insufficient to assess effects due to limited size, significant flaws, inconsistencies, gaps, or a lack of information. Further studies are needed to define the level of evidence.
**POTENTIAL BENEFITS**	
Evaluated by analyzing the balance between desirable (benefits) and undesirable (harms/burden) effects to determine the net health benefit. This involves evaluating the certainty of evidence for both beneficial and harmful outcomes separately.	
**Substantial**	There is a high chance that the net benefit is substantial.
**Moderate**	There is moderate certainty that the net benefit is moderate to substantial.
**Small**	There is likely to be only a small benefit from this therapy.
**Not known**	The evidence is lacking, of poor quality, or conflicting results, and the balance of benefits and harms cannot be determined.
**Harmful**	There is moderate or high certainty that the therapy has no benefit or that the harms outweigh the benefits.
**GRADE OF RECOMMENDATION**	
Based on several factors, including the balance of desirable versus undesirable effects (benefits versus harms), the quality of published evidence, cost-effectiveness, feasibility, societal acceptance, and the wisdom of experts.	
**A**	The committee recommends that clinicians routinely provide this therapy.
**B**	The committee recommends that clinicians consider providing this therapy to selected subjects depending on individual circumstances.
**C**	The committee concludes that the current evidence is insufficient to assess the therapy’s benefits and harms.
**D**	The committee does not recommend therapy.
**E**	Clinicians should discourage the use of this therapy.

Each statement was evaluated by an expert panel based on level of evidence, potential benefits, and grade of recommendation. A 70% consensus threshold was set for accepting evidence, benefits, and recommendations. A positive report on the vaccine had to include a level of evidence of high or moderate, a potential benefit of substantial or moderate, and recommendations of A or B.

**Table 3:** Phase I and II trials in healthy adults with the hepatitis E vaccine HEV 239 (Hecolin).

Phase	Region	Trial Objectives	Design	Dosage Schedule	No. of Subjects	ADRs	Immune Response	Conclusion	Reference
I	China	Safety Immunogenicity	Random open-label, parallel	0, 1 & 6 mon Dose: different (30 μg)	120 healthy adults 16–65 years	Local, mild, no SAEs	Immunogenic	Safe and immunogenic	[102]
II	China	Safety Immunogenicity	Randomized, controlled	Dose scheduling, dose escalation	457 adults and 155 students.	Local, mild, No SAEs	Recommended 0, 1 & 6 mon, Dose 0.5 (30 μg)	Three doses: 0, 1 & 6 mon; 0.5 m (30 μg)	[107]
II	China (2013)	Safety Immunogenicity (HBsAg +/−)	Randomized (1:1) (HEV vs. HBV vaccine)	0, 1 & 6 mon Dose: 30 μg	14,065 HBsAg + 803.	Local, 10.2%; systemic, 20%; no sig. diff. in either group; no SAEs.	Robust, +89.3 (HBsAg+) vs. 89.69 (HBsAg−)	Safety and immunogenicity for HBsAg (+) adults are very similar to those for the general population up to 2 years.	[109]
I	USA (2019)	Safety Immunogenicity	Randomized (4:1), double-blinded, placebo-controlled	Days 1, 29 & 180 Dose: 30 μg	25 healthy adults 18–45 years	Local; mild & temporary; no SAEs.	Immune response: 100%, robust	Safe and elicits a durable immune response	[108]
II/III	India	Safety Immunogenicity	Randomized double-blind	0, 1 & 6 mon	Vaccine 97; placebo 93	Mild; no SAEs	88.9 versus 22.4%	The vaccine is safe and immunogenic	[106,110]

**Table 4:** Safety, immunogenicity, and long-term efficacy of HEV-239 (Hecolin^®^) in healthy Chinese adults.

Phase	Region	Trial Objectives	Design	Dosage Schedule	No. of Subjects	ADRs	Immune Response	Results	Conclusion	Reference
**III**	Jiangsu, China (2011)	Safety Efficacy	Randomized (1:1), double-blind, placebo-controlled (HEV vs. HBV Vaccine)	0, 1 & 6 mon 30 μg Follow-up 19 months	110,165 healthy adults	Few & mild; no SAEs	-	None in the vaccine group vs. 15 in the placebo group had HEV infection	Safe & effective in the prevention of hepatitis E in the general population	[113]
III [Extended]	Jiangsu, China (2015)	Long-term efficacy	Randomized (1:1), double-blind, placebo-controlled (HEV vs. HBV Vaccine)	0, 1 & 6 mon 30 μg Follow-up 4.5 years (extended)	110,165 healthy adults	Similar between the two groups	87% maintained HEV antibodies vs. 9% in the control group at 4.5 years	Seven in the vaccine group vs. 53 in the placebo group had HEV infection	Vaccine-induced immunity lasted & protected against hepatitis E for up to 4.5 years	[111]
III [Extended]	Jiangsu, China (2024)	Long-term efficacy	Randomized (1:1), double-blind, placebo-controlled (HEV vs. HBV Vaccine)	0, 1 & 6 mon 30 μg Follow-up 10 years (extended)	110,165 healthy adults	Similarly to the two groups	87.3% maintained HEV antibodies at 8.5 years	Thirteen in the vaccine group vs. 77 in the placebo group had HEV infection (86.6%)	Vaccine-induced immunity lasted & protected against hepatitis E for up to 10 years	[112]

**Table 5:** HEV 239 (Hecolin^®^): Safety and immunogenicity in pregnancy and women of childbearing age.

Phase	Region	Trial Objectives	Design	Dosage Schedule	No. of Subjects	ADRs	Immune Response	Results	Conclusion	Reference
**IV**	Matlab, Bangladesh 2024	Safety Efficacy	Double-blind, cluster-randomized (HEV vs. HBV vaccine); 2-year follow-up	0, 1 & 6 mon	19,460 non-pregnant women (16–39), (9478 vs. 9982)	Comparable in both groups; no SARs	Miscarriage is higher in the HEV vaccine group (5.5% vs. 3.9%)	No pregnant women developed HEV infection in either group	Efficacy uncertain; higher risk of miscarriage is a matter of concern; safe with no SARs	[115]
**IV**	Matlab, Bangladesh (2024)	Safety in pregnancy	Double-blind cluster-randomized (HEV vs. HBV vaccine), follow-up at 2 years (further analysis of data)	0, 1 & 6 mon	5011 pregnant women (2407 vaccinated vs. 2604 control)		-	Miscarriage Proximal vaccination: 8.9% vs. 4.5 ** During pregnancy: 10.5% vs. 5.3% **; Distal vaccination: 5.6% vs. 4.5% (NS)	Vaccination shortly before or during pregnancy is associated with miscarriage	[120]
**IV**	Bentiu, South Sudan (2024)	Safety in pregnancy	Emulated target trial (vaccinated vs. unvaccinated pregnant women)	Vaccination before or during pregnancy; interview 2 weeks after delivery	2036 vaccinated & 638 not vaccinated	-	-	Fetal loss 7.2% vs. 6.1% (NS, risk ratio 1.2% (95% CI 0.7–1.9)	No increased risk of fetal loss in women vaccinated before or during pregnancy	[116]

** = significant, NS = not significant.

**Table 6:** HEV 239 (Hecolin^®^): safety, immunogenicity, and efficacy in CLD, including compensated cirrhosis, and chronic HBV infection.

Phase	Region	Trial Objectives	Design	Dosage Schedule	No of Subjects	ADRs	Immune Response	Results	Conclusion	References
**IV**	Shenzhen, China (2025)	Safety Immunogenicity Compensated CHB Cirrhotic	Stratified (4 groups)	0, 1 & 6 months	162 (43 CHB Cirrhosis, 50 Treated CHB, 50 untreated CHB, 19 controls)	Local, 5.26% to 24%; systemic, 0 to 12.0%. No SAEs	95% Cirrhotic seroconverted; 82.8% had optimum titers 1 month after last dose	-	Safe and immunogenic in compensated CHB cirrhosis	[122]
IV	China (2025)	Efficacy in HBV-related CLD	Test negative design	0, 1 & 6 mon 30 μg Follow-up 10 years (extended)	HEV vaccination status in 96 HEV cases vs. 2830 test-negative controls	-	-	HEV vaccination had an efficacy of 81.5% among participants in the phase III trial	The vaccine is highly effective at preventing HEV infection in patients with CHB	[123]

**Table 8:** The characteristics of immune responses to the hepatitis E virus acquired through natural infection, vaccination, or a combination of both (hybrid immunity) [141,144].

Feature	Natural Immunity (Post-Infection)	Vaccine Immunity (Post-Vaccination)	Hybrid Immunity (Infection + Vaccination)
**Source**	Prior exposure and recovery from HEV infection (often asymptomatic).	Vaccination, primarily with the Hecolin^®^ (HEV 239) recombinant vaccine available in China.	A combination of prior natural infection and subsequent vaccination.
**Efficacy against Disease**	Provides protection, estimated at around 70%, against clinically apparent hepatitis.	Highly effective, with a three-dose regimen showing over 90% efficacy in trials.	Both vaccine-induced and naturally acquired immunity significantly lower the risk of infection, suggesting strong protection when combined.
**Efficacy against Infection**	Protection is durable but can be incomplete; antibody loss is possible over time.	High levels of protection against infection were observed in clinical trials.	Expected to provide robust and potentially superior protection compared to either method alone.
**Antibody Levels**	Varies widely; often associated with modest antibody responses, especially in asymptomatic cases.	Induces a strong and measurable anti-HEV IgG antibody response, with specific target levels associated with protection.	Vaccination after natural infection significantly increases and boosts antibody levels.
**Duration of Protection**	Antibodies can persist for several years, offering durable protection.	Long-term follow-up studies suggest protection can last for at least 8.5 to 10 years.	Single-dose vaccination in individuals with pre-existing immunity achieves high and sustained antibody levels for over 103 months (approx. 8.5 years).
**Immune Mechanism**	Involves both humoral (antibodies) and cellular (T-cell) immune responses.	Primarily focuses on stimulating humoral immunity via the recombinant ORF2 capsid protein and induces T-cell responses.	Combines and enhances both arms of the immune response, leading to a potentially more comprehensive and durable immune memory.

For HEV, studies indicate that individuals with pre-existing natural immunity who are then vaccinated show highly persistent and potentially enhanced antibody responses compared with either approach alone.

**Table 10:** Limitations of expert consensus report on use of hepatitis E vaccine in India.

Limitation	Recommendations
Lack of WHO prequalification for routine use, though it is recommended for outbreak response.	Prioritize Prequalification (PQ) or WHO Emergency Use Listing (EUL), and licensing in other countries in Asia & Africa.
Lack of efficacy data outside China.	Efficacy trials need to be done in other countries, especially in Asia and Africa
Concerns about safety among pregnant women, as reported in a 2024 Bangladesh study.	Need more data about safe use in pregnant women; for example, a South Sudan study,
Uncertainty about HEV cross-genotype protection.	There is a need for a universal vaccine effective against all HEV genotypes and applicable across all regions.
Insufficient data on the safety and efficacy in individuals under 16 years of age	Safety and efficacy studies need to be done in children below 16 years of age.
Insufficient data on the safety and efficacy in immunosuppressed patients.	Safety and efficacy studies need to be done in immunosuppressed patients
Logistic and financial challenges for widespread implementation in resource-limited settings.	Vaccine availability at source should be prioritized by countries/global bodies through advance purchase agreements and stockpiling for emergency need, especially in outbreak settings.
Issue of knowledge gap and hesitancy among medical practitioners.	In-service programs, Hepatitis E updates, and drills to respond to outbreaks.
No cost-effectiveness studies have been conducted specifically in India.	While targeted vaccination for high-risk groups may be viable, universal coverage for 1.4 billion people is currently unfeasible given the high cost and three-dose schedule.

## Data Availability

The raw data used to make this consensus report are available at Dr. Khuroo’s Medical Clinic. These can be made available upon request from the expert panel members.

## Abbreviations

HEV = Hepatitis E virus; HBV = hepatitis B virus; VLP = Virus-Like-Particle; HEV-gt = Hepatitis E virus genotype; ACLF = acute-on-chronic liver failure; ACCORD = Accurate Consensus Reporting Document; COPE = Committee on Publication Ethics; ICMJE = International Committee of Medical Journal Editors; guidelines; EQUATOR = Enhancing the QUAlity and Transparency Of health Research Network guidelines; GRADE system = Grading of Recommendations Assessment; Development and Evaluation; SAGE = WHO’s Strategic Advisory Group of Experts; GACVS = the Global Advisory Committee on Vaccine Safety; CLD = Chronic Liver Disease; CFR = Case Fatality Rate; DALYs = disability-adjusted life years; (T = 1, T = 3), where T stands for “Triangulation number”; SOT = Solid organ transplant; PLWH = People living with HIV; IVI = International Vaccine Institute; IDPs = internally displaced people; WASH = water, sanitation, and hygiene.
